# Deep Removal and Photodegradation of Methylene Blue Dye Using Superabsorbent Polymer Hydrogel Composite with Activated Charcoal and TiO_2_ Nanoparticles

**DOI:** 10.1021/acsomega.4c11428

**Published:** 2025-06-16

**Authors:** Syed Sikandar Shah, Bruno Ramos, Larissa Otubo, Antonio Carlos Silva Costa Teixeira

**Affiliations:** † Research Group in Advanced Oxidation Processes, Department of Chemical Engineering, Escola Politécnica, 28133 University of São Paulo, 05508-010 São Paulo, SP, Brazil; ‡ Department of Chemical Engineering, 42511 Centro Universitário FEI, 09850-901 São Bernardo de Campo, SP, Brazil; § Nuclear and Energy Research Institute (IPEN), Av. Prof. Lineu Prestes, 2242, 05508-000 São Paulo, SP, Brazil

## Abstract

The low-cost and effective interaction of hydrogels with pollutants has drawn significant interest in the wastewater treatment domain. This study aims to explore a novel, biodegradable superabsorbent polymer (SAP) hydrogel composite incorporated with activated charcoal (AC) and TiO_2_ nanoparticles to efficiently remove and subsequently photodegrade methylene blue (MB) dye. The addition of activated charcoal boosted the adsorption capacity of the composite hydrogel, while the integration of TiO_2_ nanoparticles increased its photocatalytic activity. The adsorption reaction was allowed to proceed over 24 h, during which small sample aliquots (5 mL) were collected at specific time intervals. After the 24-h period, the MB adsorption capacity was found to be 198.99 mg g^–1^ for SAP-TiO_2_ and 203.97 mg g^–1^ for SAP-AC/TiO_2_, respectively. After a 24-h time interval of maximum MB adsorption, MB degradation was conducted by exposing the solution to a UV-A source positioned at a distance of 15 cm. The maximum MB degradation percentages under UV-A irradiation were found to be 77.11 ± 1.9 using SAP-TiO_2_ and 87.77 ± 2.9 using SAP-AC/TiO_2_, respectively. This study demonstrates the potential of a SAP composite hydrogel, incorporating AC and TiO_2_ nanoparticles, as a promising material for efficiently removing and degrading MB dye from wastewater.

## Graphical abstract

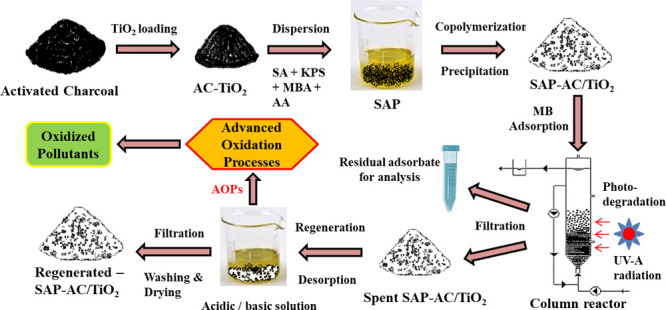

## 1 Introduction

Presently, the global issue of inadequate clean and safe water has emerged as a pressing concern. The persistent threat to clean water resources is primarily attributed to widespread industrialization and urbanization. Water bodies are being polluted by untreated waste discharged from various industries such as mining, agriculture, food processing, textile manufacturing, paper production, leather tanning, paint production, and dyeing.^1,2^ These effluents introduce toxic substances like metals, dyes, surfactants, pesticides, and other pollutants into the water.^3^ At present, there are various types of pollutants found in freshwater, including physical, chemical, organic, inorganic, biological, and radiological contaminants. Among these, dyes and trace metals are particularly harmful and pose significant environmental risks. These pollutants have the potential to negatively impact both humans and animals, necessitating their prompt and adequate attention.^4,5^

Synthetic dyes are commonly utilized to enhance the visual appeal of numerous consumer goods, such as processed foods and textiles.^6^ However, these dyes exhibit incomplete adhesion to the modified materials, resulting in their release into the environment and giving rise to significant environmental problems.^7,8^ Both cationic and anionic dyes contribute to the contamination of water bodies, rendering their removal extremely difficult. Even at trace levels, these dyes can significantly pollute vast water bodies, causing aesthetic disturbances, reducing sunlight penetration, and subsequently hindering photosynthesis. Among them, methylene blue (MB) stands out as the most prevalent and hazardous.^9^ MB is a thiazine-based cationic dye that is widely employed in the textile industry for coloring fabrics such as cotton, wool, silk, and others.^10^ It is generally characterized as nonbiodegradable, stable, highly soluble in water, and carcinogenic, posing threats to aquatic life and human health alike.^11,12^

A promising technique for eliminating MB dye from wastewater involves utilizing UV-A radiation, which falls within the range of 320–400 nm. Various factors, such as the initial dye concentration, solution pH, and UV-A radiation intensity, have been identified as influencers of the efficiency of MB dye degradation using UV-A radiation. Hence, additional research is required to optimize the conditions for achieving maximum degradation efficiency. In this study, a hybrid biodegradable superabsorbent polymer (SAP) hydrogel composite was prepared with the incorporation of activated charcoal (AC) and TiO_2_ nanoparticles for efficient adsorption and subsequent photodegradation of MB dye using UV-A radiations from simulated aqueous solution. TiO_2_ nanoparticles are widely used as photocatalysts due to their low cost, high chemical stability, low toxicity, strong oxidizing power, photostability, and unique semiconducting properties.^13^ However, their application in aqueous solutions is hindered due to separation and recovery after application and the tendency to aggregate, which limits their effectiveness as photocatalysts.^14^ In addition, TiO_2_ nanoparticles have no inherent buoyancy and tend to sink in aqueous media. In order to enhance their applicability in water treatment and enable allow doe their capability, they must be incorporated or immobilized into a suitable support matrix.^15^ To address these limitations, the incorporation of carbonaceous materials with TiO_2_ nanoparticles has been reported to enhance their performance in water treatment applications.^16^ The incorporation of AC and its engagement with the SAP hydrogel surface can establish a receptive environment for the adsorption of dye molecules. AC was chosen due to its high surface area, well-developed porosity, and excellent adsorption capacity, which makes it an ideal support material for TiO_2_ impregnation. Furthermore, AC provides a synergistic effect due to the enhanced dispersion of the TiO_2_ nanoparticles, thereby preventing their agglomeration and increasing overall photocatalytic performance. AC plays a crucial role in retaining MB molecules and accelerating their degradation when exposed to UV radiation in the presence of impregnated TiO_2_ nanoparticles. Additionally, the synthesized SAP composite hydrogel exhibits buoyancy i.e. it can float on the water surface, enabling effortless application and facilitating the photodegradation of organic pollutants in practical scenarios by remaining afloat on the water surface without sinking to the bottom.

## 2 Materials and Methods

### 2.1 Materials

Sodium alginate (SA) biopolymer (C_6_H_9_NaO_7_) with a viscosity of 15–20 cP, acrylic acid (AA, 99%) monomer, free radical initiator as a potassium persulfate (KPS), 99% (Merck), the cross-linker *N, N*-methylenebis­(acrylamide) (MBA), 99% (Aldrich), titanium dioxide (TiO_2_) nanopowder (Degussa P25), activated charcoal (AC), and methylene blue (MB) dye were acquired from Aldrich and were used without further purification. Ultrapure water (18.2 MΩ cm) from a Milli-Q system (Merck Millipore, Burlington, MA, USA) was used to prepare all the simulated dye solutions.

### 2.2 Preparation of SAP-AC/TiO_2_

To prepare AC/TiO_2_, activated charcoal was first impregnated with TiO_2_ (10 wt %) using a simple wet impregnation method. A stoichiometric amount of powdered AC was mixed with the corresponding TiO_2_ solution and heated to 60 °C for 1 h with continuous stirring. Subsequently, the mixture was dried in an oven at a temperature below 100 °C. Once completely dried, the AC/TiO_2_ composite was stored in an airtight glass vial for future use. To create the hybrid SAP-AC/TiO_2_ hydrogel, the same procedure was followed for the SAP preparation in our previous study.^2^ A predetermined SA quantity was gradually dissolved in ultrapure water under continuous stirring until complete dissolution was achieved. The resulting solution was then transferred into a four-necked jacketed reactor fitted with a mechanical stirrer, thermometer, reflux condenser, and a nitrogen (N_2_) gas inlet. The solution was stirred for 30 min, while the temperature was gradually increased to 60 °C. To eliminate dissolved O_2_, the system was purged with N_2_ flushing, after which KPS (1.5 wt %), predissolved aqueous solution, was added to the viscous mixture to initiate the formation of alginate free radicals. The mixture was subsequently stirred for a further 30 min. Separately, approximately 45 mL of AA (70% neutralized), was dispersed in 100 mL of ultrapure water. This solution was introduced dropwise to the reaction flask under a continuous N_2_ atmosphere to maintain an inert environment and to prevent premature oxidation. Moreover, after the addition of the cross-linker MBA (0.5 wt %), a slurry of AC-TiO_2_ (10 wt %) was introduced into the solution, and the temperature was increased to 70 °C and maintained for 3 h to ensure the completion of the polymerization reaction. The SA to AA ratio was carefully controlled and maintained at 1:5. The polymerized product was allowed to cool, thoroughly rinsed with ultrapure water to remove any unreacted residues, and transferred to a Petri dish for thorough drying in an oven at 70 °C. After drying, the SAP-AC/TiO_2_ hydrogel was sectioned into smaller pieces (0.5 cm) and set aside for swelling and MB adsorption/photodegradation experiments. The photographs of dried and swollen hydrogel composites have been depicted in Figure S1.

### 2.3 Column Adsorption Studies

In a continuous flow glass column with fixed dimensions (6 cm diameter and 30 cm height), initial adsorption studies were conducted. The column was filled with either 70 mg of SAP-TiO_2_ or SAP-AC/TiO_2_, and a 300 mL solution of MB dye with 50 ppm initial concentration was circulated through the column using a peristaltic pump as depicted in Figure S2. To mitigate the impact of light interference, aluminum foil was employed to cover both the column reactor and the MB solution reservoir. The optimization of the adsorption parameters was achieved using a uniform Doehlert array design for temperature and pH as variables.^2,17–19^ This optimization was facilitated using response surface methodology (RSM) with the primary aim of attaining optimal elimination of contaminants.^17^ The Doehlert design presented in Table S1 exhibits the configuration of two factors, along with their corresponding coded and real values.

Equation 1 was used to convert the coded values into their corresponding actual experimental values:

$$V_{i}=A_{i}+(X_{i}\times M_{i})$$ 1

where *V*_*i*_ represents the real experimental value, *A*_*i*_ is the average value, i.e., [(max + min)/2], *X*_*i*_ denotes the coded variable and *M*_*i*_ is the average difference, i.e., [(max–min)/2].

### 2.4 MB Photocatalytic Degradation

The photocatalytic activity of the synthesized SAP composite samples was investigated using UV-A radiation to degrade MB dye. A total of 70 mg dried composite hydrogel was retained in a glass tube reactor, and a 300 mL MB dye (50 ppm) solution was circulated through the glass reactor using a peristaltic pump at a flow rate of 28 mL min^–1^. The mixture was subjected to a 24-h adsorption process by keeping the reservoir, glass reactor, and tubing covered with aluminum foil to avoid any interference of room light. Following the completion of MB adsorption equilibrium (24 h) using the optimized adsorption conditions for each adsorbent, the column housing with the expanded polymer composite was introduced into the photoreactor. The photoreactor was irradiated by two 15-W black light UV-A lamps (Sylvania F15W/350 BL T8) emitting in the wavelength range of 300–400 nm with a maximum at 364 nm, positioned horizontally 15 cm above the transparent glass column containing the swollen polymer hydrogel. The spectral irradiance of the lamps was measured using a spectroradiometer (Luzchem, SPR-4002) as previously reported by Nunes et al.,^19^ and the corresponding calculated power density (fluence) was found to be 0.53 mW cm^–2^. The MB photodegradation process involved constant recirculation for 300 min, during which samples were collected at specific time intervals. The concentrations were spectrophotometrically measured at 665 nm, which corresponds to the maximum absorption wavelength of MB. For the complete regeneration of the spent adsorbent after the photocatalytic degradation process, the residual MB (≤10% after photocatalytic degradation) desorption process was achieved using 10 mL NaOH (0.1 mol L^–1^) solution per gram of the swollen spent adsorbent. To determine the extent of photocatalytic degradation of MB dye, a calibration curve was established and utilized. Subsequently, a UV/vis spectrophotometer set at λ_max_ = 665 nm was employed to determine the remaining concentration of MB after the photodegradation process.

### 2.5 Adsorption Kinetics

In order to determine the time at which adsorption equilibrium is reached and to gain insights into the mechanisms governing the adsorption process, the data set was employed to fit pseudo-first-order and pseudo-second-order kinetic models. The models are presented using Equations 2 and 3 individually, where *q*_*e*_ and *q*_*t*_ represent MB adsorption capacity adsorbed at equilibrium and at time *t* (mg g^–1^) respectively, and *k*_*1*_ (min^–1^) and *k*_*2*_ (g mg^–1^ min^–1^) are the rate constants for the pseudo-first-order and pseudo-second-order kinetic models, correspondingly.^20,21^

$$\ln(q_{e}-q_{t})=\ln\;q_{e}-k_{1}t$$ 2

$$\frac{t}{q_{t}}=\frac{1}{k_{2}{q_{e}}^{2}}+\frac{t}{q_{e}}$$ 3

The results of the kinetics studies were plotted to determine the rate controlling process and the MB adsorption mechanism, which are pivotal factors for real-world applications. To establish the kinetics parameters for MB adsorption, the adsorption experiments were performed over four different contact time intervals.

### 2.6 Adsorption Isotherms

To examine the adsorption isotherm, two widely recognized models namely Langmuir and Freundlich isotherm models were employed.^22^ The Langmuir adsorption model suggests monolayer coverage onto homogeneous surfaces with identical binding sites and its linear form is presented in eq 4.

$$\frac{C_{e}}{q_{e}}=\frac{1}{K_{L}q_{m}}+\frac{C_{e}}{q_{m}}$$ 4

The Freundlich isotherm model assumes multilayer adsorption on heterogeneous surfaces with adsorption sites of varying energies as expressed by eq 5.

lnqe=lnKF+1nlnCe 5

where *C*_*e*_ represents the adsorbate equilibrium concentration (mg L^–1^) in the solution, *q*_*e*_ is the adsorbate amount adsorbed at equilibrium (mg g^–1^), *q*_m_ denotes the maximum monolayer adsorption capacity (mg g^–1^), adsorption intensity is denoted by *n*, and *K*_L_ and *K*_F_ represent Langmuir and Freundlich isotherm constants, correspondingly.

### 2.7 Characterization of SAP Composites

The SAP hydrogel composite samples were characterized by HR-TEM, EDS, and XRD analysis. The high-resolution transmission electron microscopy (HRTEM) technique was used to study and observe the nanostructure of the TiO_2_ before and after the MB adsorption process. HR-TEM images were obtained with a JEOL JEM 2100 microscope operating at 200 kV. Energy-dispersive X-ray microanalysis (EDS) was performed using Field Emission Gun Scanning Electron Microscope (FEG-SEM) FEI–INSPECT F50. X-ray Diffraction (XRD) patterns were recorded using a Bruker (D8-Discover) powder X-ray diffractometer at a 2θ range of 10–100° and at a scanning rate of 0.75° min^–1^. Fourier transform infrared (FTIR) spectroscopy was carried out using a Shimadzu IRPrestige-21 spectrometer equipped with platinum diamond attenuated total reflectance (ATR). Spectral data were recorded between 4000 to 400 cm^–1^, with an accumulation of 32 scans at a resolution of 4 cm^–1^.

## 3 Results and Discussion

### 3.1 Physicochemical Characterization of SAP Adsorbents

The SAP hydrogel composite samples were characterized by HR-TEM, EDS, and XRD analysis.

#### 3.1.1 TEM Analysis

Figure 1 shows TEM micrographs of SAP-TiO_2_ and SAP-AC/TiO_2_ at various magnifications prior to MB adsorption. Figure 1a,c clearly reveals the presence of TiO_2_ nanoparticles embedded within the SA-*g*-PAA hydrogel, observed as an amorphous matrix surrounding the nanoparticles (Figure 1d). This observation is further corroborated by the existence of Ti and O peaks in the EDS spectrum of the SA-grafted-AC/TiO_2_ hydrogel composite (Figure 3). The high-resolution TEM images show crystalline planes of rutile and anatase structures, in Figure 1b,d, respectively. The presence of both phases was further confirmed by XRD analysis (Figure 4).

**Figure 1:**
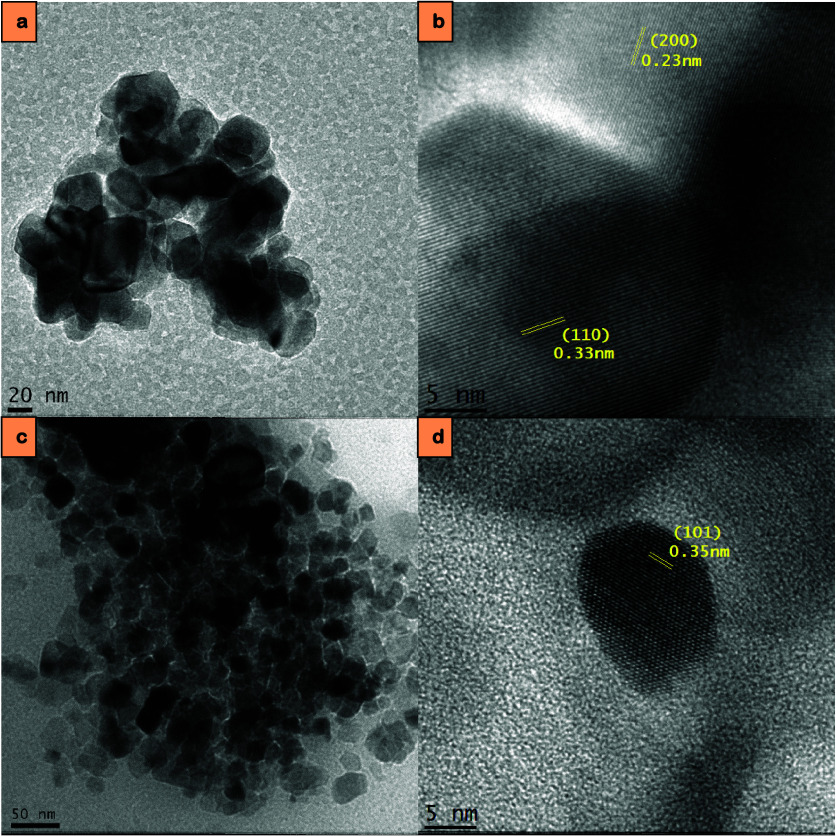
TEM micrographs of (a) SAP-TiO_2_ at 50,000× magnification, (b) SAP-TiO_2_ at 350,000× magnification, showing plane (200) of anatase and plane (110) of rutile, (c) SAP-AC/TiO_2_ at 40,000× magnification, and (d) SAP-AC/TiO_2_ at 400,000× magnification, prior to MB adsorption, showing plane (101) of anatase phase.

As observed, TiO_2_ nanoparticles within the dried hydrogel aggregate to a great extent and are generally evenly dispersed throughout its bulk. This suggests that the polymer network has a less efficient interaction with the surface of TiO_2_ nanoparticles, leading to a more pronounced aggregation within the hydrogel.

Evidently, the polymer network of the hydrogel holds the nanoparticles without forming adhesion bonds. This phenomenon enables solute molecules to potentially penetrate the photocatalytically active surface of the immobilized photocatalyst nanoparticles within the hydrogel. Subsequently, these solute molecules undergo photocatalytic decomposition under UV irradiation. Therefore, it appears that the absence of an adhesion interaction between the polymer network of the hydrogel and the photocatalyst nanoparticles is a crucial factor for their photocatalytic activity when immobilized in a hydrogel. Otherwise, the adsorbed organic polymer units themselves would likely undergo photocatalytic decomposition instead.^23^

Both materials exhibit clear signs of organized parallel meso-channels. Additionally, the TEM micrographs reveal that the TiO_2_ nanoparticles have an average particle size ranging from 20 to 40 nm. Previous research has indicated that smaller particle sizes positively influence the photodegradation of dyes and other organic pollutants, thereby enhancing the likelihood of effective photocatalytic performance against various contaminants.^24^ TEM micrographs of the SAP hydrogel composites after MB adsorption are depicted in Figure S3. No significant changes were observed in the FTIR spectra of the synthesized samples, and the obtained peaks were consistent with those typically reported in our previous studies and also in the literature for hydrogels.^2,25–28^

#### 3.1.2 EDS Analysis

An elemental mapping technique was employed to verify the existence of TiO_2_ nanoparticles within the SAP composite hydrogel. The results, depicted in Figure 2, reveal the presence of titanium (blue), oxygen (red), and carbon (purple) distributed uniformly throughout the SAP samples. Additionally, an EDS analysis of the same area confirms the presence of titanium, carbon, and oxygen, as indicated by the peaks observed in the spectrum. The weight percentages of these elements are 41.79% carbon, 46.17% oxygen, and 12.04% titanium, respectively.

**Figure 2:**
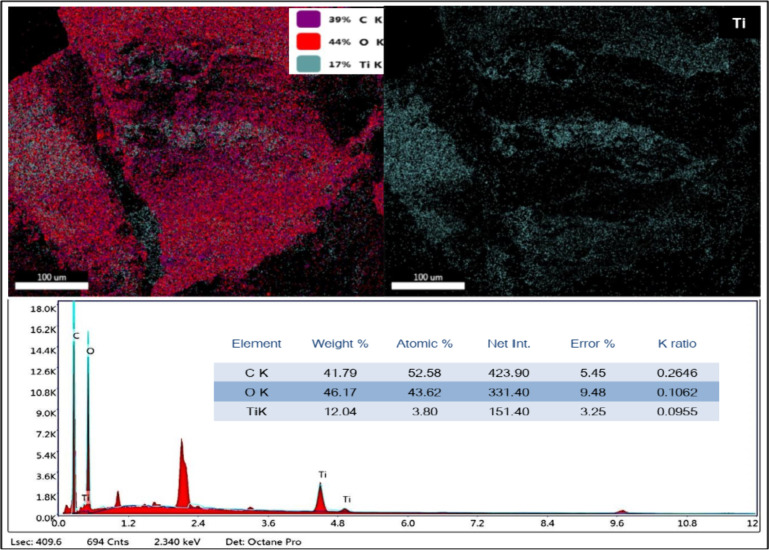
EDS analysis of the synthesized SAP-TiO_2_ composite before MB adsorption.

High-dispersion X-ray spectroscopy was employed to analyze the chemical composition of the spent SAP composite hydrogel following MB adsorption. The elemental mapping of the SAP composite matrix was performed, and Figure S4 illustrates the EDS profile of the SAP-TiO_2_ composite hydrogel after MB adsorption. The EDS spectra provide evidence of the presence of TiO_2_ nanoparticles, along with C, O, and Ca. The presence of calcium is likely due to analytical error or contamination, as the table indicates only 0.35% by weight and 0.13% atomic Ca, with a significant margin of error of 40.70%. Considering the significant level of uncertainty and the minimal amount detected, this minor signal does not impact the interpretation of the EDS results; no other element besides carbon (C), oxygen (O), and titanium (Ti) was identified in the other composite samples. Based on the EDS studies, it has been determined that even after MB adsorption and regeneration, the SAP-TiO_2_ composite hydrogel retains a sufficient and uniform distribution of TiO_2_ nanoparticles within the hydrogel matrix. Furthermore, there is no indication that the adsorption process negatively affected the presence of TiO_2_ in the SAP hydrogel.

Figure 3 presents the EDS results for the SAP-AC/TiO_2_ composite, which consists of activated charcoal impregnated with TiO_2_ nanoparticles and dispersed within the SAP polymer matrix. The weight percentages of the detected elements are as follows: carbon, 55.46%; oxygen, 39.39%; and titanium, 5.15% respectively. The EDS spectra (Figure 3) indicate the presence of both titanium and oxygen, evidencing the formation of TiO_2_ nanoparticles.^29^

**Figure 3:**
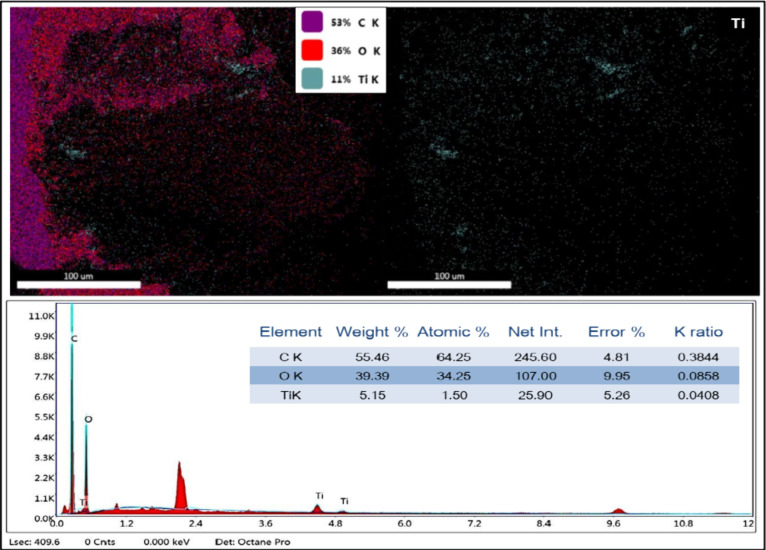
EDS analysis of the synthesized SAP-AC/TiO_2_ composite before MB adsorption.

EDS analysis of the spent SAP-AC/TiO_2_ after successful MB adsorption is displayed in Figure S5. The EDX spectrum confirms the presence of titanium and oxygen in the TiO_2_ nanoparticles, indicating the absence of impurities. Additionally, the map images of Ti reveal a uniform distribution of TiO_2_ nanoparticles throughout the SAP hydrogel matrix. Notably, there are no evident clusters or agglomerations of TiO_2_ nanoparticles, confirming the crucial role of activated charcoal in facilitating the adhesion and impregnation of nanoparticles on its surface. The weight percentages of the elements in the regenerated composite are carbon, 47.52%; oxygen, 50.59%; and titanium, 3.69%. A slight reduction in the weight percentage of TiO_2_ nanoparticles is observed after MB adsorption, due to copious amounts of washing and rinsing. This finding highlights the potential for regenerating the spent SAP-AC/TiO_2_ composite and reusing it effectively for multiple MB adsorption and desorption cycles.

#### 3.1.3 XRD Analysis

X-ray diffraction (XRD) analyses were conducted to study the composition and crystallographic characteristics of the hybrid adsorbent. XRD patterns of the synthesized samples prior to MB adsorption are presented in Figure 4. No substantial peaks were observed in the XRD pattern of SAP and SAP-AC, which confirmed their amorphous nature. However, due to the incorporation of TiO_2_ nanoparticles in SAP-TiO_2_ and SAP-AC/TiO_2_, several significant peaks appeared as shown in Figure 4, as expected. These peaks are consistent with the JCPDS Card no. 78-2486 (TiO_2_ anatase).^30^

**Figure 4:**
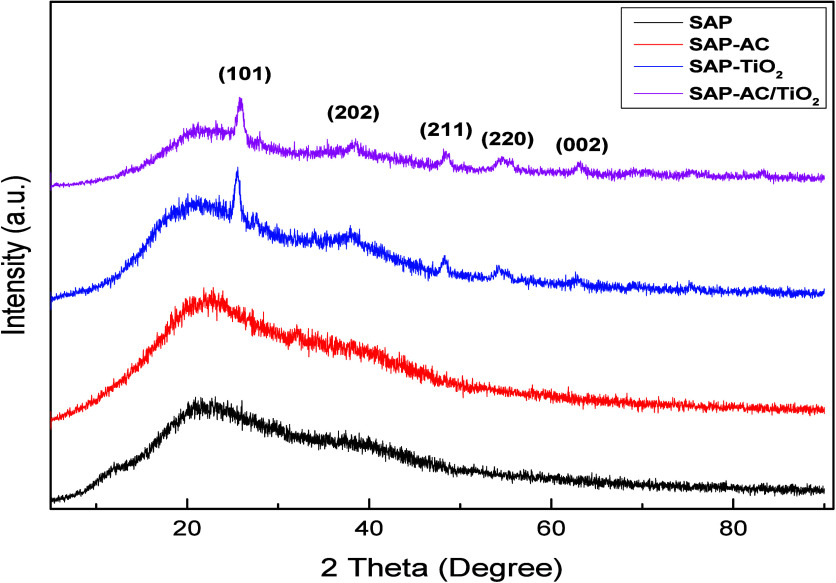
XRD pattern of SAP and its composites.

In the pattern of SAP-TiO_2_ and SAP-AC-TiO_2_, the peaks of (101), (202), and (211) are associated with the anatase phase. The characteristic peaks of (220) and (002) are attributed to the rutile phase according to JCPDS no. 12-2176,^31^ which also indicated that the percentage of the rutile phase is less than anatase phase.^25,32,33^ TiO_2_ can be found in three different polymorphs i.e. rutile, anatase, and brookite. According to the XRD analysis, rutile and anatase phases of TiO_2_ were observed in this study. Anatase has a band gap of 3.2 eV that absorbs in the UV region and has a small crystalline structure with a high surface area. The band gap for rutile is 3.0 eV with good stability and high crystal size but lower surface area. However, the coexistence of these two phases has been shown to enhance the photocatalytic performance by facilitating band gap alignment.^34–37^ The broad peak of SAP material indicated that it has poor crystallinity and having amorphous nature.^2^

#### 3.1.4 FTIR Analysis

Attenuated Total Reflectance spectroscopy (ATR) was employed to observe the Fourier-transform infrared (FTIR) absorption spectra of SAP-TiO_2_ and SAP-AC/TiO_2_ samples. Figure 5 illustrates the FTIR spectra of the synthesized samples.

**Figure 5:**
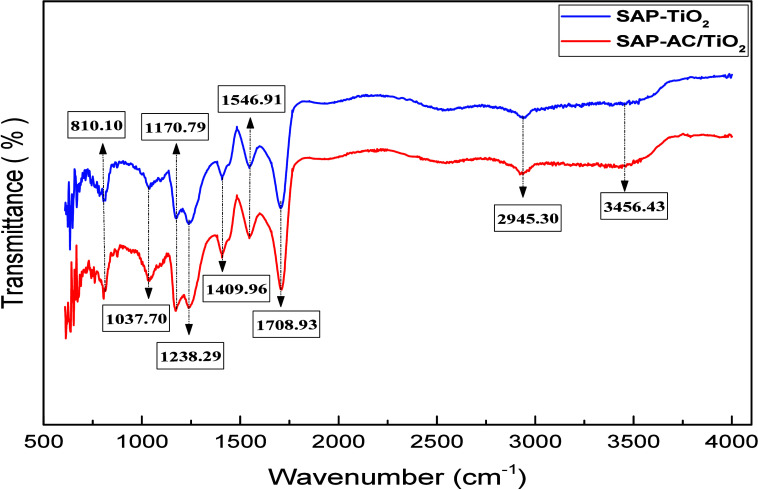
FTIR spectra of the SAP-TiO_2_ and SAP-AC/TiO_2_ composite hydrogels.

The characteristic bands observed include 810.10 and 1238.29 cm^–1^, which correspond to C=O stretching vibrations. The presence of carboxylic groups is indicated by the band at 1170.79 cm^–1^, while symmetrical stretching vibrations of carbonyl groups appear at 1409.96 cm^–1^. Additionally, the band at 1546.91 cm^–1^ is associated with the overlapping of the C=O stretching vibration of the acrylic acid unit (−C=O in–COOH). The C=O stretching of the β-carboxylic acid is observed at 1708.93 cm^–1^, while the methylene (−CH_2_−) vibrations are observed at 2945.30 cm^–1^. Finally, the broadband around 3456.43 cm^–1^ corresponds to hydroxyl bond (−OH) stretching, due to hydrogen bonding. Overall, no significant changes were observed in the FTIR spectra of the synthesized hydrogel, with the bands identified aligning well with those reported in the literature.^38–41^ Notably, the band appeared near 1708.93 cm^–1^, associated with the C=O stretching of the carboxylic group, confirming the successful grafting of acrylic acid onto sodium alginate, and validating the formation of the SAP hydrogel.^2^

### 3.2 Effect of pH on MB Adsorption at Constant Temperature

To assess experimental errors using SAP-TiO_2_ and SAP-AC/TiO_2_ as adsorbents, a series of tests were conducted. At an initial concentration of 50 ppm, MB adsorption reached approximately 98.3% with 70 mg of the adsorbent, which was attributed to the abundance of adsorption sites. However, the adsorption capacity decreased upon adding more adsorbent, leading to the determination that the optimal dosage for MB adsorption was 70 mg. This decline was due to the concentration gradient between the adsorbent and MB dye.^42^ Experiment 1, carried out at a pH of 6.0 and a temperature of 37 °C, was replicated twice. The equilibrium data for MB adsorption was computed and illustrated in Figure 6a,b against the adsorption time. The data demonstrates a gradual increase in MB adsorption over time, reaching 191.21 mg g^–1^ with SAP-TiO_2_ and 193.53 mg g^–1^ using SAP-AC/TiO_2_ after a 24-h interval. The consistency of results between these adsorbents is supported by the reproducibility of the experimental data as depicted in Figure 6a,b.

**Figure 6:**
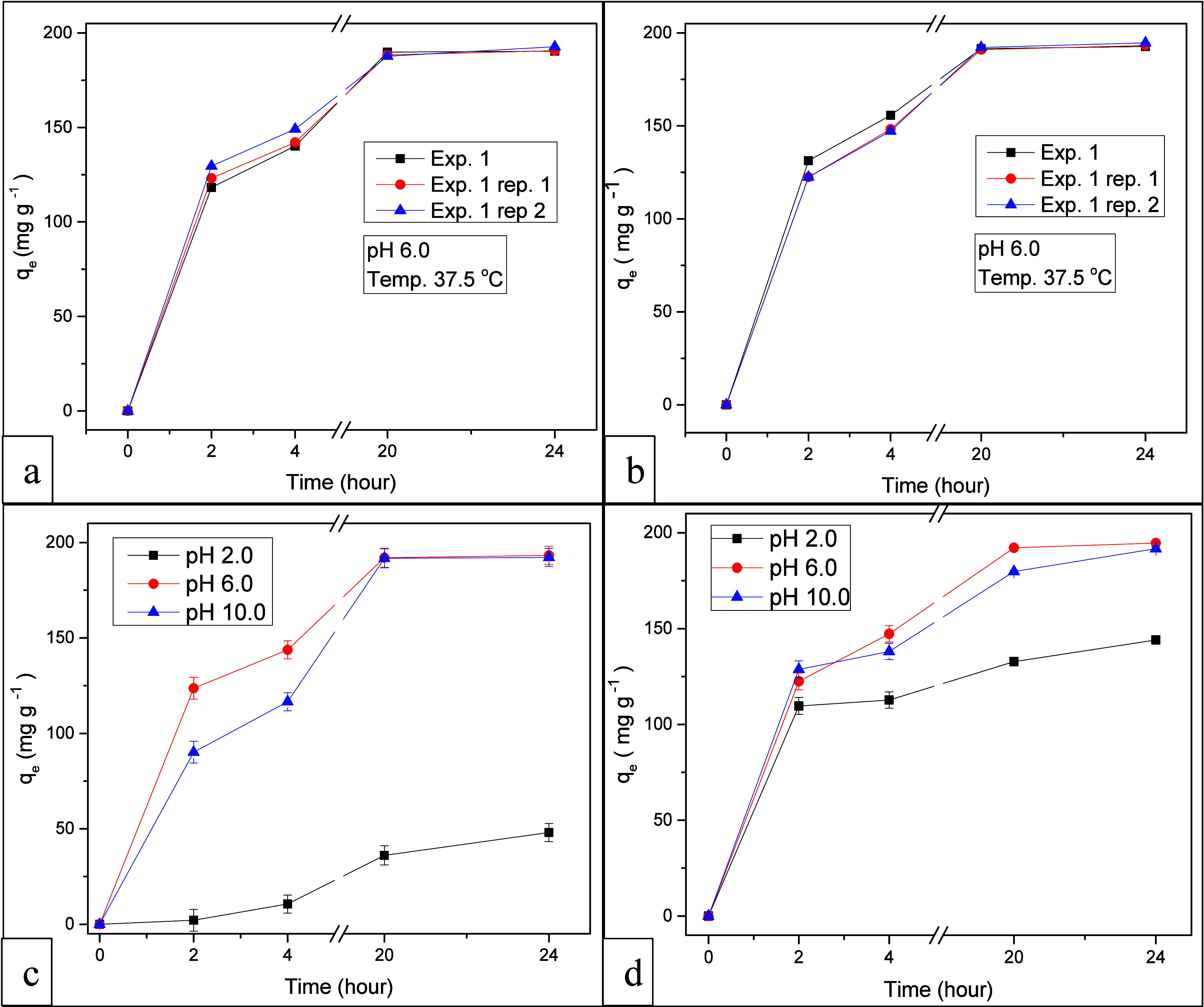
Effect of pH on MB adsorption over time using (a, c) SAP-TiO_2_ and (b, d) SAP-AC/TiO_2_ as adsorbents.

To investigate the pH effect on MB adsorption, experiments were conducted using SAP- TiO_2_ and SAP-AC/TiO_2_ as the adsorbent while maintaining a constant temperature of 37 °C. The MB adsorption equilibrium data was then determined and plotted against the adsorption time, as depicted in Figure 6c,d. The results illustrate a gradual increase in MB adsorption over time. After a 24-h interval, the adsorption capacity reached 193.21 mg g^–1^ at pH 6.0, subsequently 192.14 mg g^–1^ at pH 10.0, and at pH 2.0 it reached 48.05 mg g^–1^ using SAP-TiO_2_ as adsorbent, respectively.

pH plays a crucial role in aqueous systems as it influences the charge distribution of the adsorbent surface, leading to changes in the interactions between the adsorbent and dye molecules. The alteration in solution pH can have multiple effects, including the modification of the surface charge of the adsorbent, the degree of ionization of the adsorptive molecule, and the extent of dissociation of functional groups on the active sites of the adsorbent. In Figure 6d, the impact of pH variation on the adsorption of MB using SAP-AC/TiO_2_ as the adsorbent is depicted. The data indicates that MB adsorption is most favorable under neutral to alkaline conditions, with the highest MB adsorption equilibrium achieved at pH 6.0 (194.66 mg g^–1^), followed by pH 10.0 (191.74 mg g^–1^). This behavior can be attributed to the ionization of COO_–_ functional groups on the SAP surface, resulting in increased electrostatic attraction forces between MB and the adsorbent surface.^43^ Conversely, under acidic pH conditions (pH 2), the lowest MB adsorption (144.04 mg g^–1^) was recorded. This occurrence can be ascribed to the protonation of COO_–_ groups, resulting in a reduction of the main anion–anion (COO_–_-COO_–_) repulsive forces. Furthermore, an abundance of H_3_O^+^ ions destabilizes MB and competes with MB ions for the available adsorption sites on the SAP surface.^44^

Additional experiments were conducted to investigate the impact of temperature and pH on the adsorption of MB dye using SAP-TiO_2_ and SAP-AC/TiO_2_ as the adsorbent. Temperature is a critical factor influencing both the adsorption and desorption of the dye. Figure 7a,b illustrates MB adsorption capacities over time using SAP-TiO_2_ as an adsorbent at pH 4.0 and 8.0 individually. The data reveal that under alkaline conditions (pH 8.0), MB adsorption capability is superior compared to acidic conditions. MB adsorption capacity increases as the solution pH rises and under both temperature ranges, the alkaline conditions (pH 8.0) exhibited superior MB adsorption capability as compared to acidic conditions. At pH 4.0, MB adsorption capacity was found to be 181.03 mg g^–1^ at 25 °C and 200.95 mg g^–1^ at 50 °C correspondingly. Comparatively, MB adsorption capacity was higher at pH 8.0 reaching 187.14 mg g^–1^ at 25 °C and 198.99 mg g^–1^ at 50 °C correspondingly. Hence, as the pH of the solution was increased, the MB adsorption capacity was also enhanced. Consequently, the adsorption capacity remains relatively stable within this pH range, which is advantageous due to the prevalent basic nature of many real wastewaters.^45^

**Figure 7:**
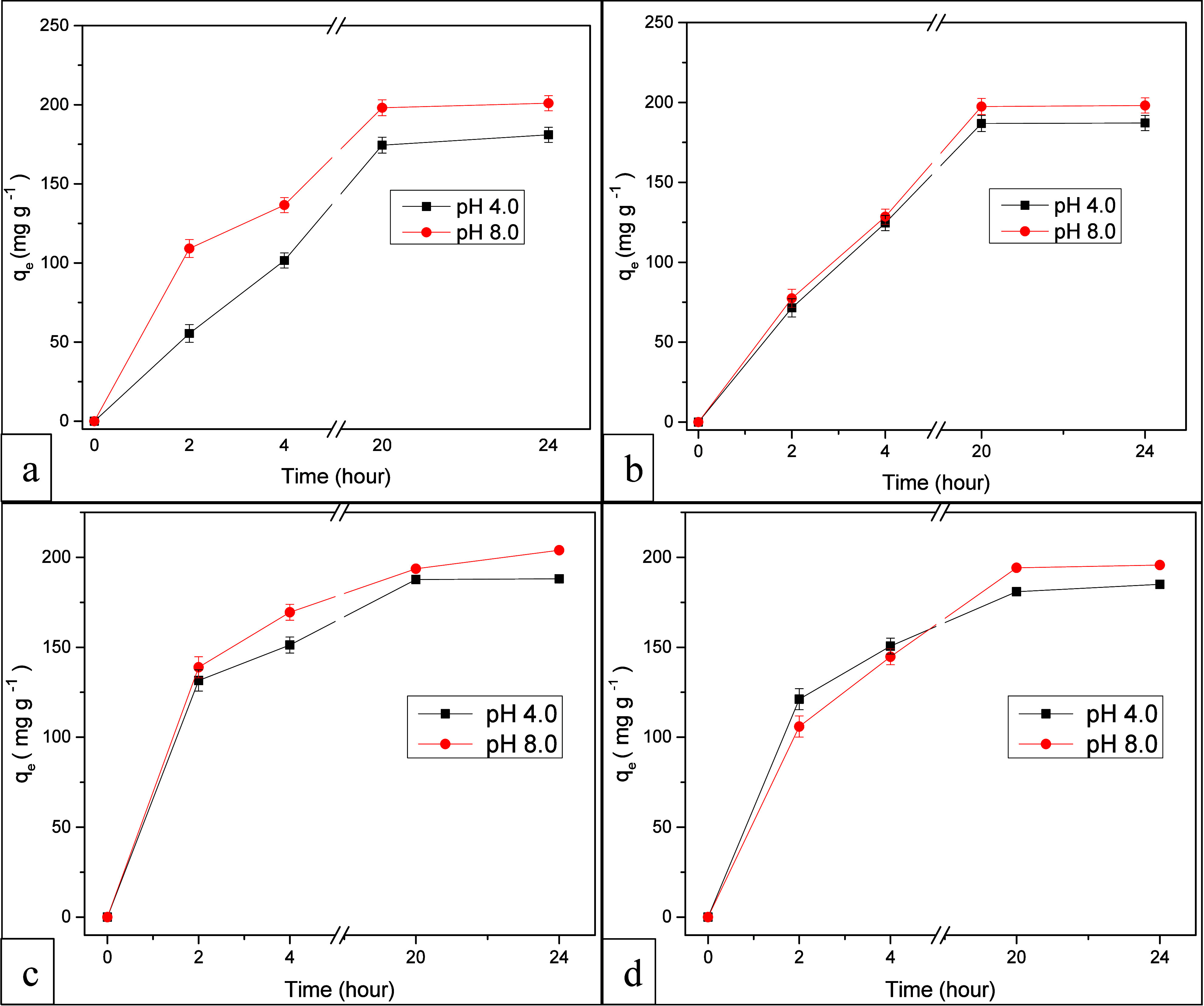
MB adsorption profiles over time at (a) 25° and (b) 50° using SAP-TiO_2_ and at (c) 25° and (d) 50° using SAP-AC/TiO_2_ as adsorbent.

Figure 7c,d shows the MB adsorption capacities at two different temperatures of 25 and 50 °C over time, comparing pH levels of 4.0 and 8.0 while utilizing SAP-AC/TiO_2_ as the adsorbent. At 25 ° temperature and at pH 4.0, MB adsorption capacity was 188.08 mg g^–1^ and at pH 8.0 it attained the value of 200.19 mg g^–1^, respectively. The obtained data revealed that under alkaline conditions (pH 8.0), the adsorption capability for MB dye was notably superior to acidic conditions, resulting in higher MB adsorption. Increasing the solution’s pH also led to an enhanced adsorption capacity of the MB dye.

Specifically, at 25 °C and pH 8.0, the adsorption capacity was found to be 203.97 mg g^–1^, while at 50 °C, it slightly decreased to 195.68 mg g^–1^. A similar trend was evident under the conditions of pH 4.0, where the adsorption of MB dye was higher (188.08 mg g^–1^) at 25 °C compared to 50 °C (184.98 mg g^–1^). This difference might be attributed to the MB dye desorption at higher temperatures.^46^ The study showed that lower temperatures (25 °C) were more favorable for MB adsorption on both SAP-TiO_2_ and SAP-AC/TiO_2_ composites. This preference for lower temperatures indicated an exothermic adsorption process. As the temperature increased, the bond between the adsorbent and MB ions weakened, causing a decrease in the adsorption capacity of MB dye. However, the impact of temperature was less pronounced compared to that exhibited by pH.

### 3.3 Effect of Temperature on MB Adsorption at Constant pH

The adsorption process can be impacted by temperature either positively or adversely. There exist two potential scenarios: first, with rising temperatures, the adsorption capacity might decline, signifying an exothermic nature of the process. Second, elevated temperature can enhance the interaction probabilities between the adsorbent and adsorbate, potentially fostering a positive direction for the adsorption process. Figure 8 shows swift adsorption during the initial few hours, followed by comparatively slower adsorption after 20-h up to 24-h. This pattern is attributed to the saturation of surface adsorption sites and the gradual diffusion of MB into the composite hydrogel porous structure. Notably, at 50 °C, SAP-TiO_2_ composite hydrogel exhibited higher and quicker equilibrium adsorption of MB dye under alkaline conditions (pH 8.0) in contrast to acidic conditions (pH 4.0), as depicted in Figure 8a,b. Moreover, the same phenomenon was observed at lower temperature conditions i.e. the MB adsorption equilibrium was achieved more rapidly at 25 °C under alkaline conditions.

**Figure 8:**
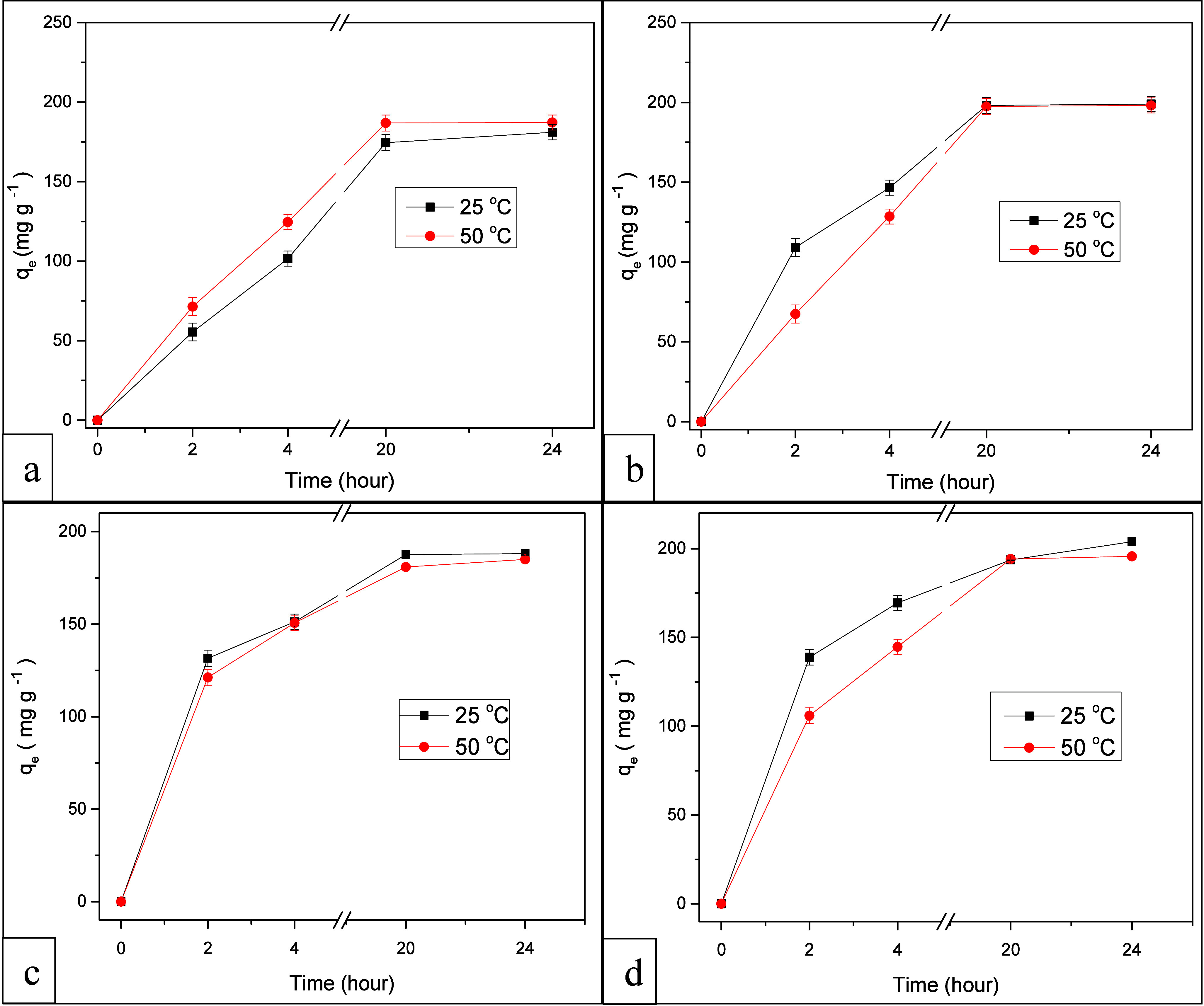
MB adsorption profiles over time at (a) pH 4.0 and (b) pH 8.0 using SAP-TiO_2_ and at (c) pH 4.0 and (d) pH 8.0 using SAP-AC/TiO_2_ as adsorbent.

The effect of temperature on the adsorption capacity of MB by the adsorbent SAP-AC/TiO_2_ was examined under two distinct temperatures (25 and 50 °C) while maintaining a constant pH. Figure 8c,d portrays the relationship between the MB adsorption capacity and time, at pH 4.0 and 8.0, respectively. When comparing the graphs, it becomes evident that the adsorption of MB by SAP-AC/TiO_2_ is more favorable at lower temperatures (25 °C) and under alkaline conditions (pH 8.0). This observation suggests that the process of MB adsorption onto SAP-AC/TiO_2_ is exothermic and the adsorption capacity diminishes as the temperature rises from 25 to 50 °C, regardless of the pH (4.0 and 8.0), as illustrated in Figure 8c,d.

Under acidic conditions (pH 4.0), maximum MB adsorption capacities were determined to be 188.08 mg g^–1^ at 25 °C and 184.98 mg g^–1^ at 50 °C respectively. Conversely, at alkaline conditions (pH 8.0), a higher capacity for MB adsorption was observed, specifically 203.97 mg g^–1^ at 25 °C and 195.68 mg g^–1^ at 50 °C using SAP-AC/TiO_2_ as adsorbent. The increased adsorption of MB dye under alkaline conditions can be attributed to the enhanced anionic surface charge of the SAP adsorbent, resulting from the deprotonation of carboxyl functional groups. This, in turn, leads to a greater number of available adsorption sites. Additionally, higher temperatures cause a weakening of the bond between the adsorbent and MB ions, contributing to a reduction in the effectiveness of MB removal once adsorbed.^47^

### 3.4 MB Adsorption Percentage and Swelling Ratio

The effect of pH and temperature on the swelling behaviors of SAP-TiO_2_ and SAP-AC/TiO_2_ composite hydrogel during the MB adsorption was investigated under various experimental conditions. Figure 9a illustrates the MB adsorption percentage and Figure 9b demonstrates the swelling ratio of the SAP hydrogel composites. The results indicated that the maximum MB adsorption percentage was attained at pH 8.0 and a temperature of 25 °C. This suggests that under alkaline conditions, the electrostatic interaction between the negatively charged carboxyl groups in SAP-AC/TiO_2_ and the positively charged MB molecules enhanced the adsorption capacity. Conversely, a decrease in MB adsorption was observed under acidic conditions, attributed to the electrostatic repulsion between the MB molecules and the adsorbent. This repulsion occurred due to the increased concentration of hydrogen ions in the solution. Consequently, the interaction between the cationic dye MB and the positively charged adsorbent diminished.^43^ Additionally, based on existing literature, it is reasonable to propose that the hydrogen bond between the hydrogel and MB weakens as the temperature rises above 50 °C. Consequently, this weakening of the hydrogen bond leads to a reduction in MB removal efficiency.^43^ In our previous study, we investigated the adsorption performance of MB dye using plain SAP and SAP-AC hydrogel composite and obtained 96.96 and 99.48% MB adsorption, respectively.^2^

**Figure 9:**
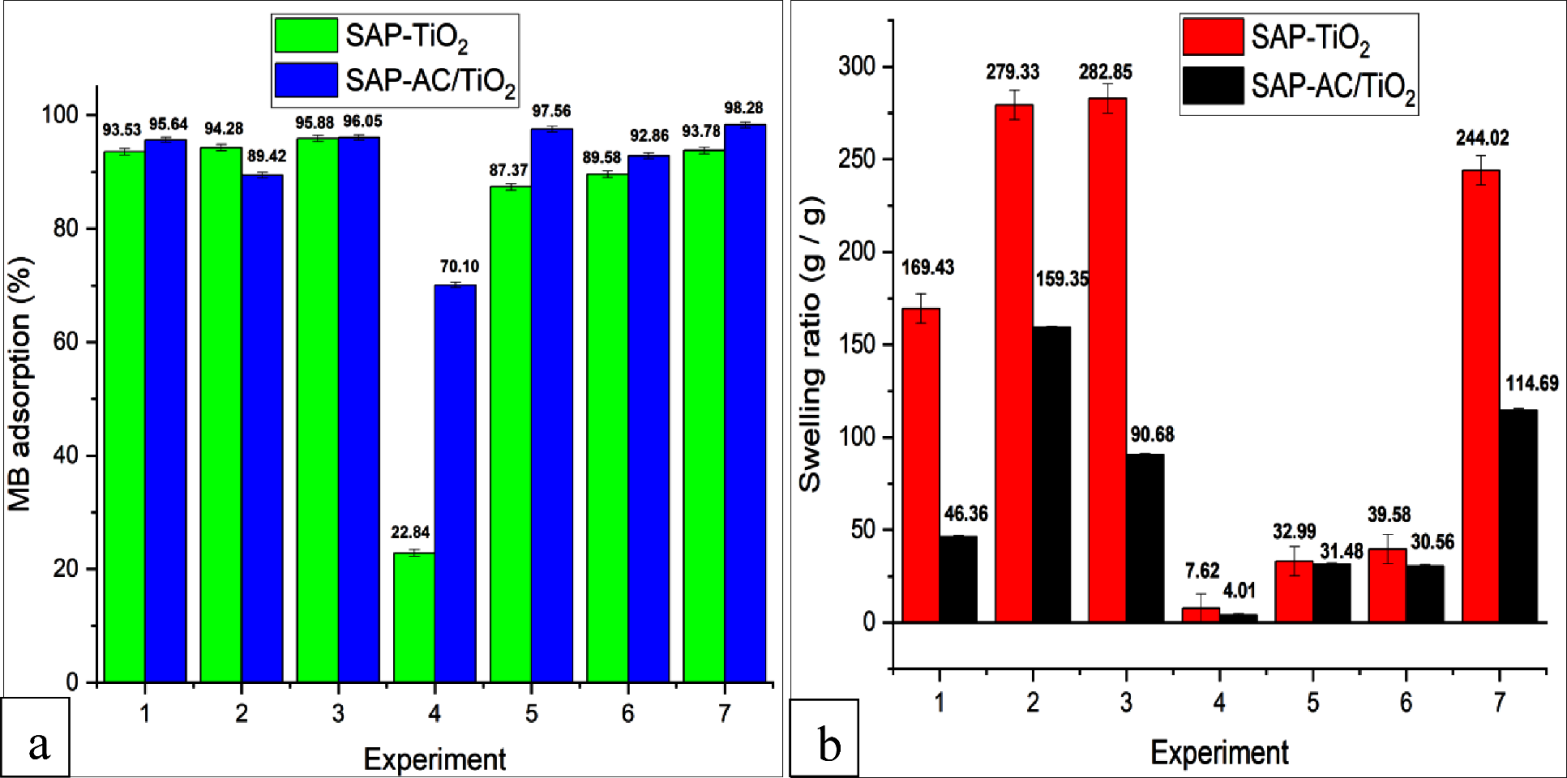
Evaluation of reaction parameters influencing (a) MB adsorption percentage and (b) swelling behavior in SAP hydrogel composites.

The swelling rate and structural stability of the hydrogels are notably influenced by the pH of the solution. In this present investigation, it was noted that elevating the pH leads to a proportional increase in the amount of water absorbed by the hydrogel, consistent with prior research findings.^48,49^ The reduced swelling ratio of SAP hydrogel composite in acidic environments (pH 2.0) may be attributed to the protonation of carboxylate anions, which leads to a reduction in repulsive forces between anions. As a result, the swelling ratio is diminished.^50^ Moreover, protonation leads to the reinforcement of H-bonds in −COOH, thereby increasing the physical cross-linking within the structural framework of the SAP molecule. In contrast, under alkaline pH conditions, the −COOH groups become ionized, causing a gradual weakening of the H-bonds.^41^ As a result, the electrostatic repulsion creates additional space within the hydrogel, allowing it to accommodate more water and significantly enhancing its swelling capacity.

### 3.5 Adsorption Kinetics

Predicting accurate adsorption kinetics is essential for designing industrial adsorption columns. In the current study, two commonly applied kinetic models, specifically, pseudo-first-order and pseudo-first-order equations were used for MB adsorption using SAP-TiO_2_ as adsorbent as shown in Figure S6.

From the plots it can be deduced that the pseudo-second-order model best fits the current data, assuming nondissociating molecular adsorption of MB molecules on SAP-TiO_2_. The values determined from the experimental data are provided in Table 1.

**Table 1:** Kinetics Parameters for MB Adsorption on SAP Adsorbents

	pseudo-first-order parameters		pseudo-second-order parameters	
SAP-TiO_2_	*q*_e_ (mg g^–1^)	121.26	*q*_e_ (mg g^–1^)	212.76
	*K*_1_ (min^–1^)	0.008	*K*_2_ (mg g^–1^ min^–1^)	0.0024
	*R* ^2^	0.969	*R* ^2^	0.996
SAP-AC/TiO_2_	*q*_e_ (mg g^–1^)	148.95	*q*_e_ (mg g^–1^)	227.27
	*K*_1_ (min^–1^)	0.007	*K*_2_ (mg g^–1^ min^–1^)	0.0015
	*R* ^2^	0.978	*R* ^2^	0.992

MB adsorption mechanism on SAP-AC/TiO_2_ was elucidated by applying two kinetic models, i.e., pseudo-first-order and pseudo-second-order equations. Both models were fitted to the experimental data for evaluation as shown in Figure S7.

Based on the pseudo-second-order kinetic model, the predicted experimental value for the amount of MB adsorbed at equilibrium *q*_*e*_ was nearly equal to the calculated value and the regression value (*R* ^2^) was also higher as compared to the pseudo-first-order model as provided in Table 1. The measured experimental *q*_*e*_ value was 203.97 mg g^–1^ compared with the calculated value of 227 mg g^–1^, which confirms that the pseudo-second-order model best fits the current data as compared to the pseudo-first-order equation.

The results indicated that the pseudo-second-order model provided a better fit for the system suggesting that the adsorption kinetics was primarily governed by chemisorption, with a rate-controlling step involving electrostatic attraction between the positively charged cationic dye molecules and negatively charged polymer chains, respectively.^26,27^

### 3.6 Adsorption Isotherms

Adsorption isotherms are of great importance in studying the adsorption behavior, capacity, and affinity of an adsorbent. Figure S8 shows the Langmuir and Freundlich adsorption isotherm models applied to the MB adsorption using SAP-TiO_2_ as an adsorbent. It is evident from the correlation coefficient (*R* ^2^ = 0.999) that the current data best fits the Freundlich model as compared to the Langmuir model. The Freundlich isotherm model assumes that the concentration of adsorbate on the adsorbent surface rises with increasing the adsorbate concentration in the solution, while the sorption energy exponentially declines as the available adsorption sites become occupied.^28^

Langmuir and Freundlich isotherm parameters and their constants are provided in Table 2. As the value *n* is greater than 1, it can be assumed that MB adsorption on SAP-TiO_2_ is favorable.

**Table 2:** Isotherm Parameters of MB Adsorption on SAP Composite Adsorbents

	Langmuir isotherm parameters		Freundlich isotherm parameters	
SAP-TiO_2_	*q*_m_ (mg g^–1^)	1048.98	*n*	1.821
	*K*_L_	0.094	*K*_F_	132.12
	*R* ^2^	0.953	*R* ^2^	0.999
SAP-AC/TiO_2_	*q*_m_ (mg g^–1^)	1149.42	*n*	1.633
	*K*_L_	0.087	*K*_F_	121.89
	*R* ^2^	0.978	*R* ^2^	0.999

Figure S9 shows the plotted version of the Langmuir and Freundlich isotherm model using SAP-AC/TiO_2_ as an adsorbent. Results are evaluated and the applicability of the model can be generalized by comparing the correlation coefficient (*R* ^2^) of their linear fit. From the plotted data, it can be deduced that the Freundlich model provides a better fit to the experimental data compared to the Langmuir model. The Freundlich isotherm is frequently applicable to adsorption on heterogeneous surfaces, assuming the presence of numerous and diverse adsorption sites acting simultaneously, each characterized by a distinct sorption free energy.^28^ Since SAP was impregnated with AC and TiO_2_ nanoparticles, the hybrid adsorbent had a number of heterogeneous sites available for MB adsorption.

The corresponding parameters and constants of the Langmuir and Freundlich model were calculated from the straight-line equation and are tabulated in Table 2. Once again, the value *n* was found to be greater than 1, which suggests a higher MB adsorption on SAP-AC/TiO_2_.

Table 3 presents a comparison of the adsorption capacity of MB as observed in this study with those reported in prior literature. It is evident that the MB adsorption capacities obtained in this study exhibit enhanced efficiency. Furthermore, the maximum MB adsorption achieved was 98.28%, utilizing SAP-AC/TiO_2_ as the adsorbent at an initial MB concentration of 50 ppm.

**Table 3:** Comparative Evaluation of Different Adsorbents for MB Uptake

adsorbent	initial MB concentration (mg L^–1^)	MB adsorption capacity (mg g^–1^)	reference
carboxymethyl cellulose/PAA/GO	100	138.4	^51^
arabic gum/PAA/PAM	50	48	^52^
nano-TiO_2_/MWCNT/chitosan	300	80.65	^53^
TiO_2_-fly ash geopolymer	100	103.19	^54^
TiO_2_-guar gum	200	184.32	^55^
TiO_2_/CQDs/alginate	30	44.13	^56^
SAP-TiO_2_	50	198.99	current study
SAP-AC/TiO_2_	50	203.97	current study

### 3.7 MB Adsorption Capacity

The experimental arrangement outlined in Table S1 was employed to investigate how pH (X_1_) and temperature (X_2_) influence the adsorption capacity of both SAP-TiO_2_ and SAP-AC/TiO_2_, utilizing Response Surface Methodology (RSM). The impact of these factors was assessed by observing the percentage of adsorption removal (*R*%), calculated after a 24-h period. The graphical representation of the response surface pertaining to the removal of MB dye using SAP-TiO_2_ as the adsorbent is depicted in Figure 10. The response surface indicates a zone of maximum removal, around neutral-to-basic pH (7–9), regardless of the temperature. In acid pH values, it appears that the temperature also shows some curvature, suggesting that temperatures near the central point (∼38) promote higher removal degrees than the extremities. The plot of predicted versus observed values indicates the validity of the model. The lower values of *R* ^2^ (= 0.88) are reflected in the discrepancy found at higher values. This suggests that the accuracy of the model could be increased if the space sample had more intermediate points (between 20 and 90%, for instance). The concentration of points near 90–100% somehow limits the capability of the model to be more representative.

**Figure 10:**
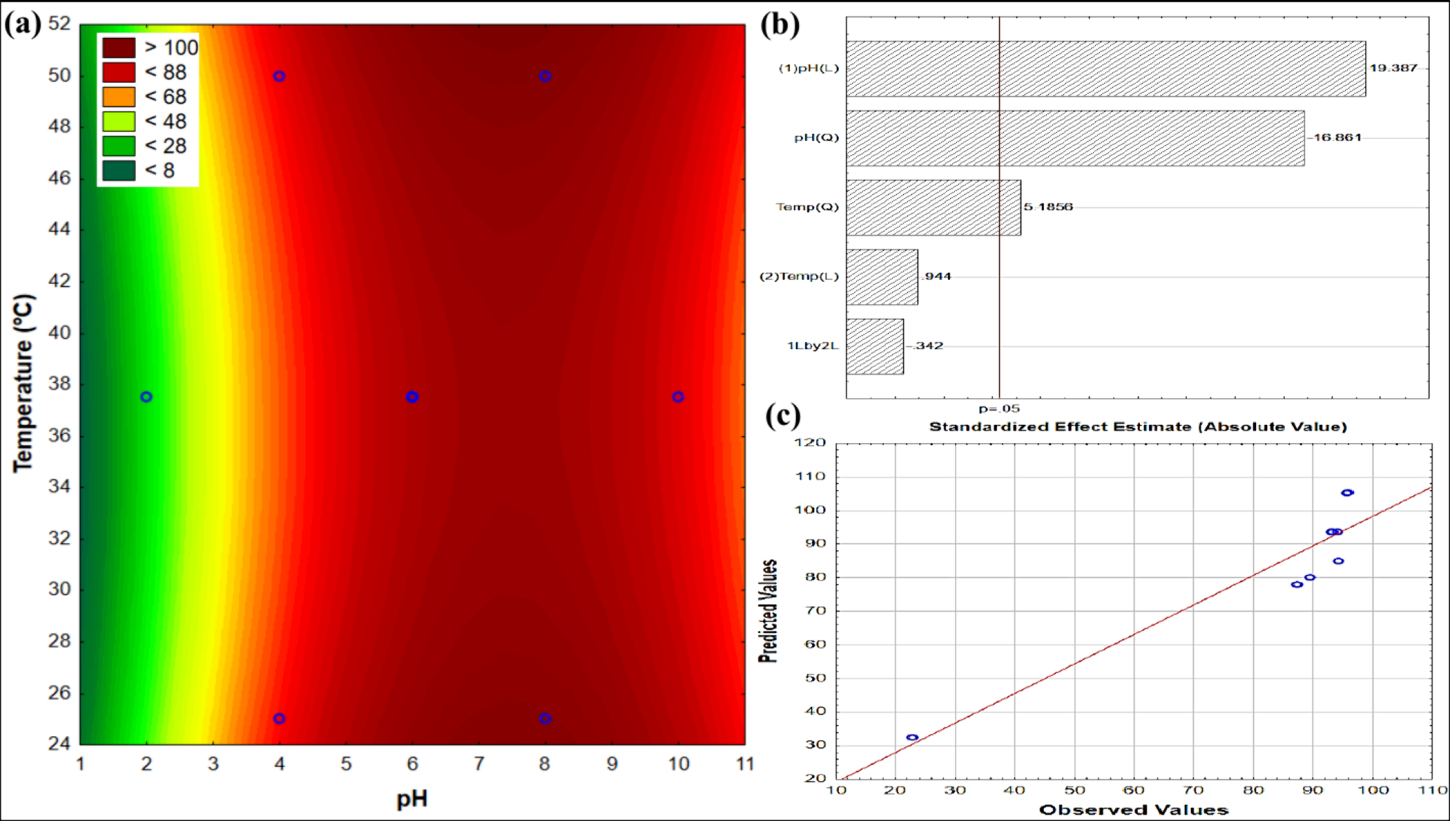
Response surface for MB adsorption percentage using SAP-TiO_2_ over 24 h continuous recirculation. (a) Contour plot depicting adsorption variation with pH and temperature; (b) Pareto chart displaying standardized effect estimates; and (c) residual plot comparing observed and predicted values (*C*_MB,0_ = 50 mg L^–1^; *V* = 300 mL; *m*_SAP‑TiO2,0_ = 70 mg).

The desirability of the MB adsorption under different temperatures and pH conditions was also monitored. Figure S10 displays the desirability of the MB adsorption using SAP-TiO_2_ as an adsorbent. Here, the desirability was defined as 1 = maximum *q*_*e*_, and 0 = minimum *q*_*e*_. The desirability is maximum within a 10% range, in the range represented by the two blue lines on the top as depicted in Figure S10. This means that we can achieve maximum *q*_*e*_ regardless of the temperature (all sampled points lie within the region limited by the blue lines), but should operate at pH levels between 6 and 8, respectively.

The response surface showing the percentage MB removal by SAP-AC/TiO_2_ over 24-h continuous recirculation is illustrated in Figure 11. The surface shows a saddle point structure, with a region of maximum removal around neutral pH levels, between 6 and 8. As a feature of saddle points, the temperature effects seem to shift between lower (and higher) pH levels, and in the optimum zone. Around neutral pH, the model indicates that lower and higher temperatures should provide better results than middle-point temperatures. However, as the middle-point results are close to 100%, this could not be reliable (we cannot have *R* > 100 in reality). Maybe one reason for the better adjustment is the spread of observed values. Here we can see that the observed R% is more dispersed than in the previous case.

**Figure 11:**
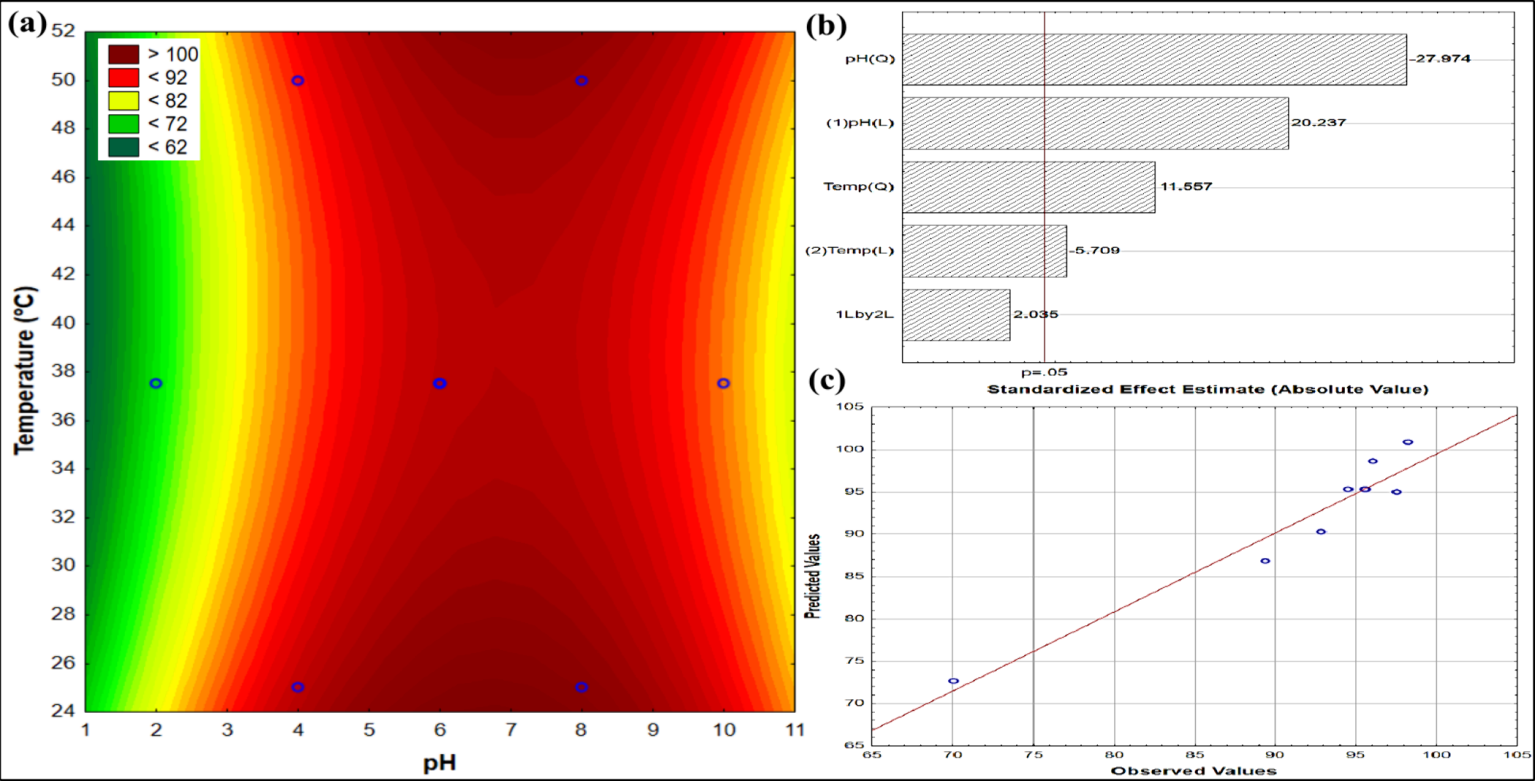
Response surface for MB adsorption percentage using SAP-AC/TiO_2_ over 24h continuous recirculation. (a) Contour plot depicting adsorption variation with pH and temperature; (b) Pareto chart showing standardized effect estimates; and (c) residual plot comparing observed and predicted values (*C*_MB,0_ = 50 mg L^–1^; *V* = 300 mL; *m*_SAP‑AC/TiO2,0_ = 70 mg).

Figure S11 shows the desirability of MB adsorption under specific temperature and pH conditions using SAP-AC/TiO_2_ as an adsorbent. The surface shows a saddle point structure, with a region of maximum removal around neutral pH levels, between 6 and 8. As a feature of saddle points, the temperature effects seem to shift between lower (and higher) pH levels, and in the optimum zone. Around neutral pH, the model indicates that lower and higher temperatures should provide better results than middle-point temperatures. However, doubts remain on this behavior, since even around neutral pH almost all of the adsorbate was removed. The desirability is similar to the observed in SAP-TiO_2_: no clear effect of the temperature, but important to use pH between 6 and 8.

### 3.8 MB Adsorption and Degradation Using UV Radiation

The study further investigated the photodegradation of MB dye utilizing SAP-TiO_2_ and SAP-AC/TiO_2_, as shown in Figure 12. It is evident that when TiO_2_ nanoparticles were absent in the SAP matrix, the degradation of MB was nearly negligible. As the irradiation time increased, the concentration of MB dye consistently decreased, indicating successful MB degradation. The maximum degradation of MB was achieved after 300 min of irradiation, prompting the cessation of the reaction by turning off the UV lamp. For comparison, plain SAP hydrogel was also evaluated for MB dye photodegradation. As observed, plain SAP does not possess any photocatalytic activity, and the negligible or minimal amount of degradation of MB dye was caused by autophotolysis (direct photolysis) by MB dye absorbing UV-A radiation directly. Direct photolysis of MB dye under UV-A radiation alone is typically slow and incomplete (9.42% after 270 min), as the energy provided by UV-A photons is insufficient to induce significant degradation. To achieve the true photocatalytic degradation efficiency of MB dye using SAP-TiO_2_ or SAP-AC/TiO_2_, the influence from direct photolysis was accounted for and deducted from the overall degradation measurements at specific time intervals. The SAP-AC/TiO_2_ hydrogel composite demonstrated its highest photocatalytic degradation efficiency for MB dye by achieving a degradation rate of 87.77% at 240 min of degradation time. Comparing SAP-TiO_2_ and SAP-AC/TiO_2_, the latter exhibited slightly higher MB degradation (13.82%). This improvement can be attributed to the presence of activated charcoal (AC) within the SAP matrix, which helped retain MB molecules and facilitated their degradation under UV radiation. Unlike the poly­(acrylic acid) polymer network, which does not interact with the surface of TiO_2_ nanoparticles in the composite hydrogel, the presence of AC and its interaction with the hydrogel surface created an open environment for the adsorption of dye molecules. This, in turn, made the photocatalytic decomposition of the dye molecules on the surface of the TiO_2_ nanoparticles more probable, as they were effectively immobilized within the bulk of the hydrogel.^23^ The calculated apparent first-order degradation rate constants of MB were 0.036 min^–1^ for SAP-AC/TiO_2_, 0.023 min^–1^ for SAP-TiO_2_ and 0.002 min^–1^ for SAP. The MB photodegradation rate, indicated by the relative concentration (*C*/*C*_0_) over time, is illustrated in Figure 12.

**Figure 12:**
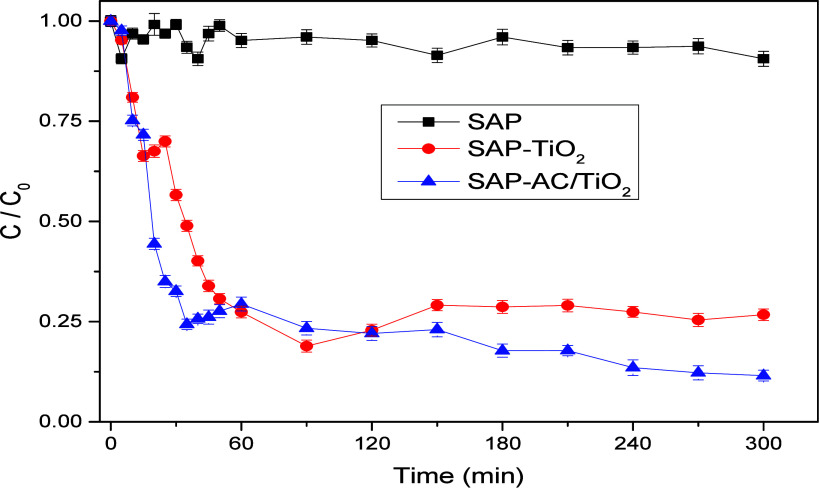
Photocatalytic degradation of MB under UV-A irradiation over time.

In this study, an effective and economical technique was employed to remove MB dye from a simulated water sample. One of the approaches utilized was the photodegradation of MB through the application of UV-A radiation. This method capitalizes on the ability of certain chemical compounds, when exposed to UV radiation in the presence of a photocatalyst, to trigger chemical reactions that result in the breakdown of organic compounds. The use of UV radiation for the photodegradation of MB dye has garnered significant attention due to its high efficiency, ease of operation, and cost-effectiveness. The process involves the absorption of UV radiation by MB molecules, leading to the generation of reactive oxygen species (ROS) like hydroxyl radicals (•OH) and singlet oxygen (^1^O_2_) as depicted in Figure S12. These ROS then target the aromatic rings and amino groups within the MB molecules, causing the cleavage of the chromophore and the conversion of MB into simpler and less toxic compounds. To further enhance the photodegradation of MB using UV radiation, the inclusion of photocatalysts like titanium dioxide (TiO_2_) is recommended. These catalysts play a crucial role in increasing the production of ROS, thereby promoting more efficient degradation of MB dye.^57,58^ Furthermore, SAP hybrid catalysts can undergo degradation over time, particularly those containing biodegradable components. Biodegradation can lead to the breakdown of the polymer matrix and catalyst components into smaller, less harmful substances, reducing their environmental impact.

Previously, researchers have explored the utilization of SA-TiO_2_ hybrid material as a potential substitute for traditional adsorbents.^59^ Thakur and Arobita^59^ investigated a hydrogel composed of cross-linked sodium alginate incorporated with TiO_2_ and found that it exhibited a remarkable maximum adsorption capacity of 1156.1 mg g^–1^ for methyl violet (MV), exhibiting an adsorption capacity as high as 99.6% when compared to SA-derived hydrogel (85%). This enhancement was ascribed to the existence of TiO_2_ within the hybrid hydrogel, which acted as an anionic center, facilitating electrostatic attraction with the MV dye. Likewise, Reveendran and Ong^60^ demonstrated the effectiveness of the SA–TiO_2_ hybrid film in the degradation of Congo red (at a concentration of 5 mg L^–1^ and pH 8) under UV radiation for 6-h, while still maintaining substantial catalytic activity even after two consecutive reuse cycles. Generally, the presence of TiO_2_ was found to be favorable for the adsorptive interaction and photocatalytic degradation of dye molecules. However, it was observed that at high dye concentrations, the photocatalytic properties of TiO_2_ could be inhibited due to surface saturation. This occurred due to the absorption of energy (light and photons) by the dye molecules, leading to a reduction of hydroxyl radicals and reactive oxygen species generation.^61^ In the current study, a higher dye concentration was used (50 mg L^–1^) and the photocatalytic properties of TiO_2_ nanoparticles embedded in the SAP matrix were not inhibited.

### 3.9 Regeneration and Recycling of the Spent Hydrogel

Effective regeneration and successive recycling of the spent adsorbent is of paramount importance for industrial applications. In this study, the spent hydrogel after MB adsorption and subsequent photocatalytic degradation was filtered and washed with pure water. For complete regeneration of the spent adsorbent, the desorption of residual MB (≤10% after photocatalytic degradation) was carried out using 10 mL NaOH (0.1 mol L^–1^) per gram of the swollen spent adsorbent. For each successful regeneration cycle, the spent adsorbent was treated with NaOH (0.1 mol L^–1^) solution for 1 h at room temperature. The solution was then neutralized and the regenerated composite hydrogel was collected, washed with pure water, and dried for further reuse. The adsorption and subsequent photocatalytic degradation cycle was repeated three consecutive times to evaluate the reusability of the adsorbent. After three consecutive MB degradation cycles, a slight decrease in photocatalytic activity was observed possibly due to the irreversible adsorption of degradation products and the biodegradable nature of the SAP material. The results of reusability experiments are shown in Figure 13. The potential for multicycle reuse is demonstrated by the consistent degradation efficiency in three consecutive cycles of MB adsorption and subsequent photodegradation. These results show the potential application of the prepared composite hydrogel for dye removal in wastewater treatment.

**Figure 13:**
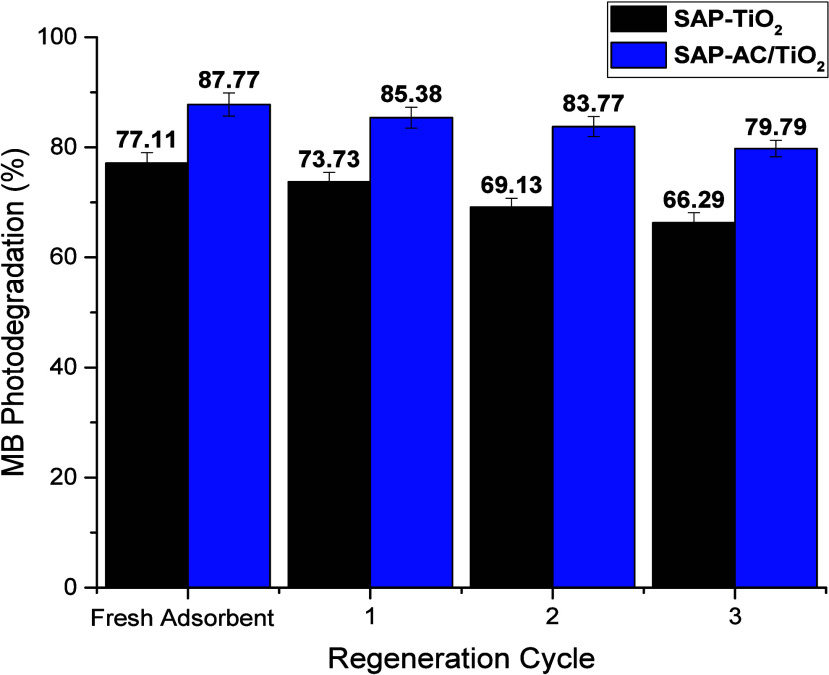
MB degradation percentage profile during regeneration cycles.

## 4 Conclusions

The synthesized SAP hydrogel composite incorporated with activated charcoal impregnated with TiO_2_ nanoparticles was found to be an efficient adsorbent and cost-effective photocatalyst for the adsorption and subsequent degradation of the MB dye. RSM analysis indicated that the optimal operating conditions for MB adsorption were pH levels between 6 and 8, regardless of temperature. The adsorption capacity was more influenced by pH than by temperature. The adsorption capacity of the SAP-AC/TiO_2_ composite hydrogel was enhanced by the introduction of AC, and its photocatalytic activity was achieved by the incorporation of TiO_2_ nanoparticles. FTIR analysis confirmed the grafting reaction of acrylic acid onto the hydrogel, and EDS analysis verified the uniform distribution of TiO_2_ nanoparticles within the hydrogel matrix. The presence of TiO_2_ nanoparticles in the SAP matrix enhanced the photocatalytic degradation of MB, with SAP-AC/TiO_2_ showing greater degradation than SAP-TiO_2_. The highest photocatalytic degradation of the MB dye was observed at a degradation time of 240 min, reaching 87.8% with the use of the SAP-AC/TiO_2_ hydrogel composite. SAP-AC/TiO_2_ exhibited a 13.8% improvement in MB degradation compared to SAP-TiO_2_. The results suggested that UV-A irradiation in the presence of photocatalysts can effectively degrade MB, indicating a potential method for water treatment.

In conclusion, this study has provided valuable insights into the adsorption behavior of MB dye using SAP-based adsorbents under different pH and temperature conditions. In addition, the floating photocatalysts enable in situ solar remediation, allowing direct treatment of contaminated wastewater reservoirs in remote locations without the need for specialized equipment or facilities. The results demonstrated the potential of SAP-TiO_2_ and SAP-AC/TiO_2_ composite hydrogels for effective MB adsorption and photodegradation.

## Supplementary Material



## Data Availability

The data of this article have been included in this manuscript.

## Notes

Doehlert design table, photographs of the studied SAP materials, schematic representation of MB adsorption setup, TEM Micrographs, EDS analyses, adsorption kinetics, adsorption isotherms, MB adsorption desirability, and schematic representation of MB photodegradation mechanism (PDF)

## References

[1] Khodakarami M., Bagheri M. (2021). Recent advances in synthesis and application of polymer
nanocomposites for water and wastewater treatment. J. Clean. Prod..

[2] Shah S. S., Ramos B., Teixeira A. C. S. C. (2022). Adsorptive
Removal of Methylene Blue
Dye Using Biodegradable Superabsorbent Hydrogel Polymer Composite
Incorporated with Activated Charcoal. Water
2022, Vol. 14, Page 3313.

[3] Vilela D., Parmar J., Zeng Y., Zhao Y., Sánchez S. (2016). Graphene-Based
Microbots for Toxic Heavy Metal Removal and Recovery from Water. Nano Lett..

[4] Ma L., Wang Q., Islam S. M., Liu Y., Ma S., Kanatzidis M. G. (2016). Highly Selective and Efficient Removal
of Heavy Metals
by Layered Double Hydroxide Intercalated with the MoS4 2- Ion. J. Am. Chem. Soc..

[5] Ma L., Islam S. M., Liu H., Zhao J., Sun G., Li H., Ma S., Kanatzidis M. G. (2017). Selective and Efficient Removal of
Toxic Oxoanions of As­(III), As­(V), and Cr­(VI) by Layered Double Hydroxide
Intercalated with MoS4 2-. Chem. Mater..

[6] Dotto G. L., Moura J. M., Cadaval T. R. S., Pinto L. A. A. (2013). Application of
chitosan films for the removal of food dyes from aqueous solutions
by adsorption. Chem. Eng. J..

[7] Dotto G. L., Lima E. C., Pinto L. A. A. (2012). Biosorption
of food dyes onto Spirulina
platensis nanoparticles: Equilibrium isotherm and thermodynamic analysis. Bioresour. Technol..

[8] Zhou Z., Lin S., Yue T., Lee T.-C. (2014). Adsorption of food dyes from aqueous
solution by glutaraldehyde cross-linked magnetic chitosan nanoparticles. J. Food Eng..

[9] Muniyandi M., Govindaraj P., Bharath Balji G. (2021). Potential removal of Methylene Blue
dye from synthetic textile effluent using activated carbon derived
from Palmyra (Palm) shell. Mater. Today Proc..

[10] Kasinathan M., Thiripuranthagan S., Sivakumar A. (2020). Fabrication of sphere-like Bi2MoO6/ZnO
composite catalyst with strong photocatalytic behavior for the detoxification
of harmful organic dyes. Opt. Mater. (Amst)..

[11] Peng Y., Zhang Y., Tian F., Zhang J., Yu J. (2017). Structure
Tuning of Bi2MoO6 and Their Enhanced Visible Light Photocatalytic
Performances. Crit. Rev. Solid State Mater.
Sci..

[12] Yu H., Jiang L., Wang H., Huang B., Yuan X., Huang J., Zhang J., Zeng Yu G. H., Jiang L., Wang H., Huang B., Yuan X., Huang J., Zhang J., Zeng G., Yu H. (2019). Modulation of Bi2MoO6-Based
Materials for Photocatalytic Water Splitting and Environmental Application:
a Critical Review. Small.

[13] Chen X., Mao S. S. (2007). Titanium dioxide nanomaterials: Synthesis,
properties,
modifications and applications. Chem. Rev..

[14] Chong M. N., Jin B., Chow C. W. K., Saint C. (2010). Recent developments in photocatalytic
water treatment technology: A review. Water
Res..

[15] Machado L. C. R., Torchia C. B., Lago R. M. (2006). Floating photocatalysts
based on
TiO2 supported on high surface area exfoliated vermiculite for water
decontamination. Catal. Commun..

[16] Bartl M. H., Puls S. P., Tang J., Lichtenegger H. C., Stucky G. D. (2004). Cubic mesoporous frameworks with
a mixed semiconductor
nanocrystalline wall structure and enhanced sensitivity to visible
light. Angew. Chemie - Int. Ed..

[17] Ferreira S. L. C., Dos Santos W. N. L., Quintella C. M., Neto B. B., Bosque-Sendra J. M. (2004). Doehlert matrix: A chemometric tool
for analytical chemistry - Review. Talanta.

[18] Doehlert D. H. (1970). Uniform
Shell Designs. Appl. Stat..

[19] Nunes R. F., Metolina P., Teixeira A. C. S. C. (2021). Dodecylpyridinium
chloride removal
by persulfate activation using UVA radiation or temperature: experimental
design and kinetic modeling. Environ. Sci. Pollut.
Res..

[20] Tan I. A. W., Hameed B. H., Ahmad A. L. (2007). Equilibrium and kinetic studies on
basic dye adsorption by oil palm fibre activated carbon. Chem. Eng. J..

[21] Ho Y. S., McKay G. (1998). Sorption of dye from
aqueous solution by peat. Chem. Eng. J..

[22] Bansal, R. C. ; G, M. ; Bansal, R. C. ; Goyal, M. Activated Carbon Adsorption. CRC Press Inc.: Boca Raton, FL, 2005.

[23] Mansurov R.
R., Safronov A. P., Lakiza N. V., Beketov I. V. (2017). Photocatalytic Activity
of Titanium Dioxide Nanoparticles Immobilized in the Polymer Network
of Polyacrylamide Hydrogel. Russ. J. Appl. Chem..

[24] Mobeen
Amanulla A., Sundaram R. (2019). Green synthesis of TiO2 nanoparticles
using orange peel extract for antibacterial, cytotoxicity and humidity
sensor applications. Mater. Today Proc..

[25] Mallakpour S., Sadaty M. A. (2016). Thiamine hydrochloride
(vitamin B1) as modifier agent
for TiO2 nanoparticles and the optical, mechanical, and thermal properties
of poly­(vinyl chloride) composite films. RSC
Adv..

[26] Bakhshi H., Darvishi A. (2016). Preparation and evaluation of hydrogel composites based
on starch-g-PNaMA/eggshell particles as dye biosorbent. Desalin. Water Treat..

[27] Cheng H.-L., Feng Q.-H., Liao C.-A., Liu Y., Wu D.-B., Wang Q.-G. (2016). Removal of methylene blue with hemicellulose/clay
hybrid
hydrogels. Springer.

[28] Vijayakumar G., Tamilarasan R., Dharmendirakumar M. (2011). Adsorption, Kinetic, Equilibrium
and Thermodynamic studies on the removal of basic dye Rhodamine-B
from aqueous solution by the use of natural adsorbent perlite. J. Mater. Environ. Sci..

[29] Bopape D. A., Tetana Z. N., Mabuba N., Motaung D. E., Hintsho-Mbita N. C. (2023). Biosynthesis
of TiO2 nanoparticles using Commelina benghanlensis for the photodegradation
of methylene blue dye and antibiotics: Effect of plant concentration. Results Chem..

[30] Wu J. J., Yu C. C. (2004). Aligned TiO2 Nanorods and Nanowalls. J. Phys.
Chem. B.

[31] Padmini M., Balaganapathi T., Thilakan P. (2022). Rutile-TiO2: Post heat treatment
and its influence on the photocatalytic degradation of MB dye. Ceram. Int..

[32] Mallakpour S. (2017). Production,
characterization, and surface morphology of novel aromatic poly­(amide-ester-imide)/functionalized
TiO2 nanocomposites via ultrasonication assisted process. Polym. Bull..

[33] Jin Y. S., Kim K. H., Choi H. W., Park S. J., Kim J. H. (2010). Properties
of TiO2 Films Prepared for Use in Dye-sensitized Solar Cells by Using
the Sol-gel Method at Different Catalyst Concentrations. J. Korean Phys. Soc..

[34] Marra M., Dumont M., Palhares H. G., Alcamand H. A., Houmard M., Nunes E. H. M. (2022). Structural and photocatalytic properties of sol–gel-derived
TiO2 samples prepared by conventional and hydrothermal methods using
a low amount of water. J. Sol-Gel Sci. Technol..

[35] Hanaor D. A. H., Sorrell C. C. (2011). Review of the anatase
to rutile phase transformation. J. Mater. Sci.
2010 464.

[36] Mikrut P., Kobielusz M., Indyka P., Macyk W. (2020). Photocatalytic
activity
of TiO2 polymorph B revisited: physical, redox, spectroscopic, and
photochemical properties of TiO2­(B)/anatase series of titanium dioxide
materials. Mater. Today Sustain..

[37] Varadwaj P. R., Dinh V. A., Morikawa Y., Asahi R. (2023). Polymorphs of Titanium
Dioxide: An Assessment of the Variants of Projector Augmented Wave
Potential of Titanium on Their Geometric and Dielectric Properties. ACS Omega.

[38] Wang W., Ni J., Chen L., Ai Z., Zhao Y., Song S. (2020). Synthesis
of carboxymethyl cellulose-chitosan-montmorillonite nanosheets composite
hydrogel for dye effluent remediation. Int.
J. Biol. Macromol..

[39] Makhado E., Pandey S., Modibane K. D., Kang M., Hato M. J. (2020). Sequestration
of methylene blue dye using sodium alginate poly­(acrylic acid)@ZnO
hydrogel nanocomposite: Kinetic, Isotherm, and Thermodynamic Investigations. Int. J. Biol. Macromol..

[40] Binma-ae H., Prasertsan P., Choorit W. (2020). Preparation and Characterization
of Biopolymers Recovered from Palm Oil Mill Effluent and Their Complex
Hydrogels Compared to Commercial Xylan. Waste
Biomass Valorization.

[41] Thakur S., Arotiba O. A. (2018). Synthesis, swelling
and adsorption studies of a pH-responsive
sodium alginate–poly­(acrylic acid) superabsorbent hydrogel. Polym. Bull..

[42] Nandi B. K., Goswami A., Purkait M. K. (2009). Removal
of cationic dyes from aqueous
solutions by kaolin: Kinetic and equilibrium studies. Appl. Clay Sci..

[43] Ravi, Pandey L. M. (2019). Enhanced adsorption
capacity of designed bentonite and alginate beads for the effective
removal of methylene blue. Appl. Clay Sci..

[44] Kong Y., Zhuang Y., Han Z., Yu J., Shi B., Han K., Hao H. (2019). Dye removal by eco-friendly
physically cross-linked
double network polymer hydrogel beads and their functionalized composites. J. Environ. Sci. (China).

[45] Alver E., Metin A. Ü., Brouers F. (2020). Methylene blue adsorption
on magnetic
alginate/rice husk bio-composite. Int. J. Biol.
Macromol..

[46] Momina, Mohammad S., Suzylawati I. (2020). Study of the
adsorption/desorption of MB dye solution using bentonite adsorbent
coating. J. Water Process Eng..

[47] Shah S. S., Jamroz N. U., Sharif Q. M. (2001). Micellization
parameters and electrostatic
interactions in micellar solution of sodium dodecyl sulfate (SDS)
at different temperatures. Colloids Surfaces
A Physicochem. Eng. Asp..

[48] Saito H., Taguchi T., Aoki H., Murabayashi S., Mitamura Y., Tanaka J., Tateishi T. (2007). pH-responsive
swelling
behavior of collagen gels prepared by novel crosslinkers based on
naturally derived di- or tricarboxylic acids. Acta Biomater..

[49] Khare A. R., Peppas N. A. (1995). Swelling/deswelling of anionic copolymer
gels. Biomaterials.

[50] He G., Ke W., Chen X., Kong Y., Zheng H., Yin Y., Cai W. (2017). Preparation
and properties of quaternary ammonium chitosan-g-poly­(acrylic
acid-co-acrylamide) superabsorbent hydrogels. React. Funct. Polym..

[51] Hosseini H., Zirakjou A., McClements D. J., Goodarzi V., Chen W. H. (2022). Removal
of methylene blue from wastewater using ternary nanocomposite aerogel
systems: Carboxymethyl cellulose grafted by polyacrylic acid and decorated
with graphene oxide. J. Hazard. Mater..

[52] Paulino A. T., Guilherme M. R., Reis A. V., Campese G. M., Muniz E. C., Nozaki J. (2006). Removal of
methylene blue dye from an aqueous media
using superabsorbent hydrogel supported on modified polysaccharide. J. Colloid Interface Sci..

[53] Parlayıcı Ş., Pehlivan E. (2024). Methylene
blue removal using nano-TiO2/MWCNT/Chitosan
hydrogel composite beads in aqueous medium. Chemosphere.

[54] Alahmad J., BiBi A., Al-Ghouti M. A. (2024). Application of TiO2-loaded fly ash-based
geopolymer in adsorption of methylene blue from water: Waste-to-value
approach. Groundw. Sustain. Dev..

[55] Santoso S. P., Angkawijaya A. E., Bundjaja V., Hsieh C. W., Go A. W., Yuliana M., Hsu H. Y., Tran-Nguyen P. L., Soetaredjo F. E., Ismadji S. (2021). TiO2/guar gum hydrogel composite
for adsorption and photodegradation of methylene blue. Int. J. Biol. Macromol..

[56] Taghiloo B., Shahnazi A., Nabid M. R. (2024). Construction
of nanocomposite hydrogel
by TiO2-carbon quantum dots encapsulated in alginate with a highly
efficient adsorption and photodegradation of dye pollutants. J. Alloys Compd..

[57] Yuan Q., Yang Y., Wu W., Dai X., Zhong J., Jian Y., Li R., Wang T., Yu H., Xia X. (2023). Synthesis of a novel TiO2/HA/RGO composite material
with photocatalytic
activity for dye degradation. Mater. Chem. Phys..

[58] Kaur H., Kumar S., Kaushal S., Badru R., Singh P. P., Pugazhendhi A. (2023). Highly customized
porous TiO2-PANI nanoparticles with
excellent photocatalytic efficiency for dye degradation. Environ. Res..

[59] Thakur S., Arotiba O. (2018). Synthesis, characterization and adsorption
studies
of an acrylic acid-grafted sodium alginate-based TiO2 hydrogel nanocomposite. Adsorpt. Sci. Technol..

[60] Reveendran G. a/p, Ong S. T. (2018). Application of experimental design
for dyes removal
in aqueous environment by using sodium alginate-TiO2 thin film. Chem. Data Collect..

[61] Anaya-Esparza L. M., de la Mora Z. V., Ruvalcaba-Gómez J. M., Romero-Toledo R., Sandoval-Contreras T., Aguilera-Aguirre S., Montalvo-González E., Pérez-Larios A. (2020). Use of Titanium Dioxide (TiO2) Nanoparticles as Reinforcement
Agent of Polysaccharide-Based Materials. Processes.

