# Supplementary material for: Deep Removal and Photodegradation of Methylene Blue Dye Using Superabsorbent Polymer Hydrogel Composite with Activated Charcoal and TiO2 Nanoparticles
Source: ACS Omega. 2025 Jun 16;10(25):26441–57. doi: 10.1021/acsomega.4c11428 (PMC12223825; doi:10.1021/acsomega.4c11428)
Supplement: Supplementary file 1 [file ao4c11428_si_001.pdf]

Deep removal and photodegradation of methylene blue dye using superabsorbent polymer hydrogel composite with activated charcoal and TiO_2 nanoparticles

SYED SIKANDAR SHAH[1*], BRUNO RAMOS[1,2], LARISSA OTUBO[3], ANTONIO CARLOS SILVA COSTA TEIXEIRA[1*],

[1] Research Group in Advanced Oxidation Processes, Department of Chemical Engineering, Escola Politécnica, University of São Paulo, 05508-010, São Paulo, SP, Brazil
[2] Department of Chemical Engineering, Centro Universitário FEI, 09850-901, São Bernardo de Campo, SP, Brazil
[3] Nuclear and Energy Research Institute (IPEN), Av. Prof. Lineu Prestes, 2242, 05508-000, São Paulo, SP, Brazil.
*E-mail: syed.shah@unesp.br , acscteix@usp.br

Supplementary Information

Table S1: Doehlert design for two factors.

Coded Values			Real values		
Exp. no.	pH	Temperature	Exp. no.	pH	Temperature (°C)
1	0	0	1	6.0	37.5
*1 rep. 1	0	0	1 rep. 1	6.0	37.5
**1 rep. 2	0	0	1 rep. 2	6.0	37.5
2	1	0	2	10.0	37.5
3	0.5	0.866	3	8.0	50.0
4	–1	0	4	2.0	37.5
5	–0.5	–0.866	5	4.0	25.0
6	–0.5	0.866	6	4.0	50.0
7	0.5	–0.866	7	8.0	25.0

*1 rep. 1 = Experiment # 1 replicated once, **1 rep. 2 = Experiment # 1 replicated twice

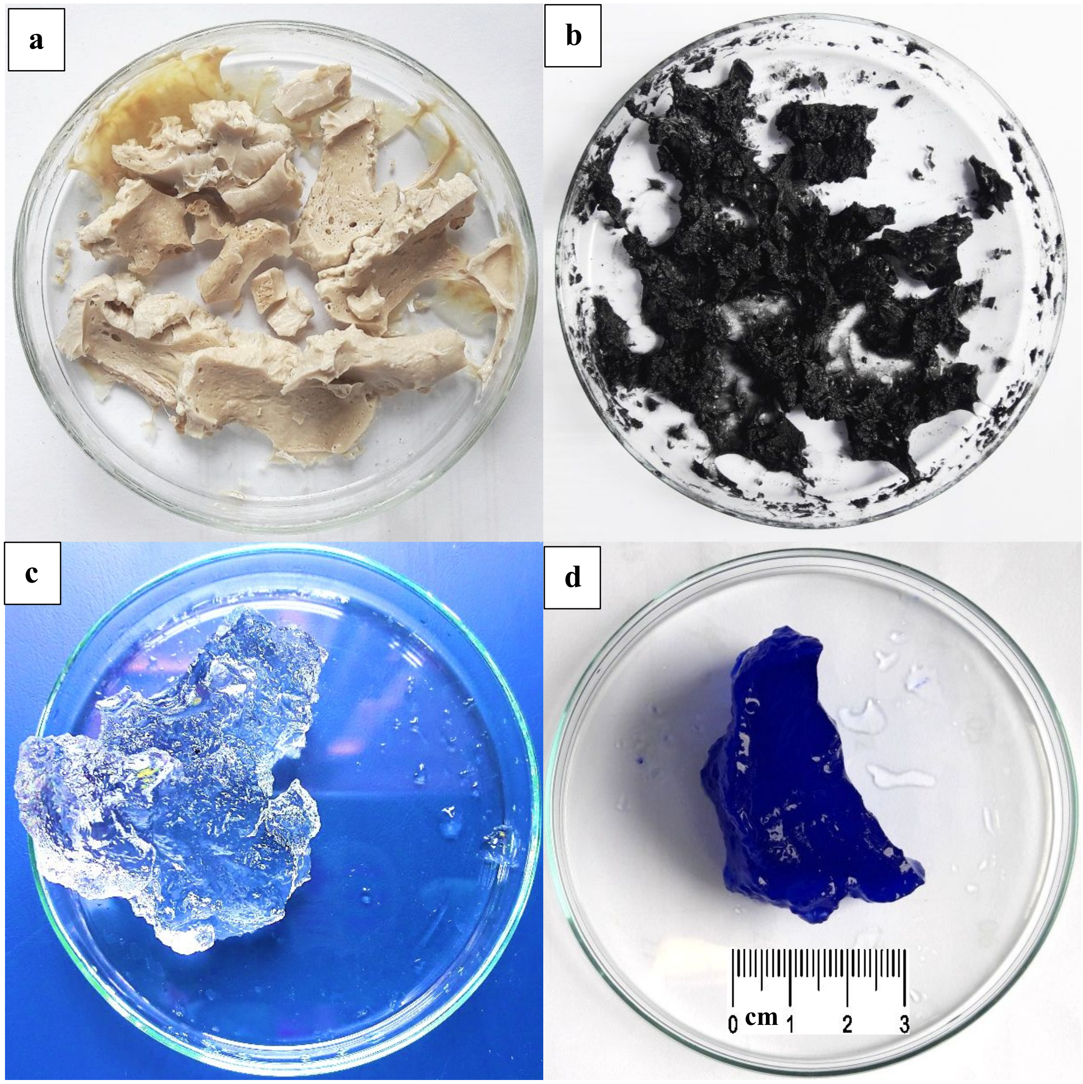

Figure S1: Photographs of the materials studied (a) SAP-TiO_2 (b) SAP-AC/TiO_2 in its dry form and (c) SAP-AC/TiO_2 after water absorption, and (d) SAP-AC/TiO_2 after MB adsorption as a swollen hydrogel.

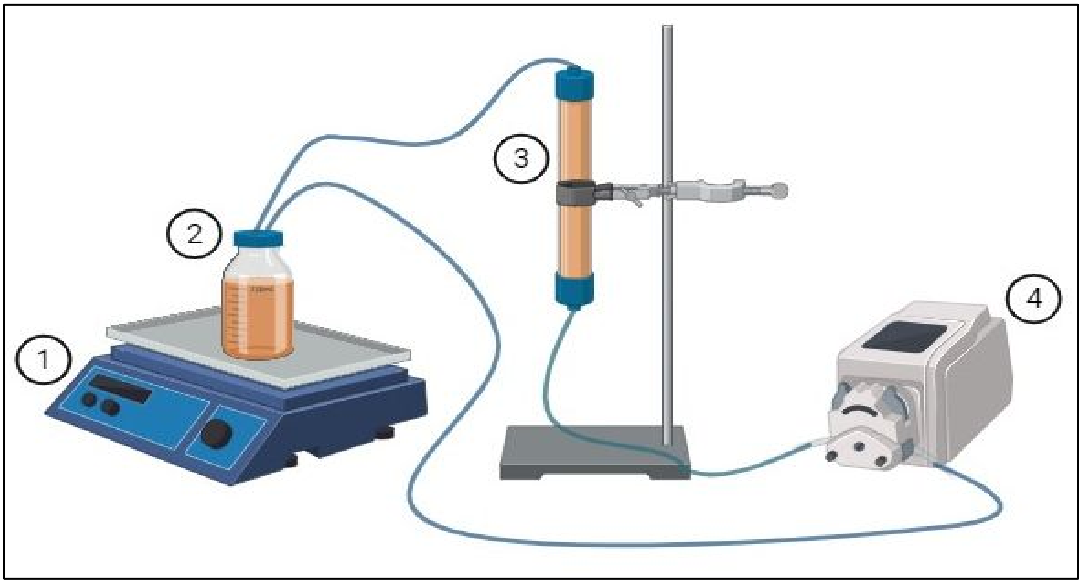

Figure S2: Schematic representation of the methylene blue (MB) adsorption setup. 1: Heating plate stirrer; 2: Reservoir with circulating MB solution; 3: Glass column reactor; 4: Peristaltic pump for solution recirculation.

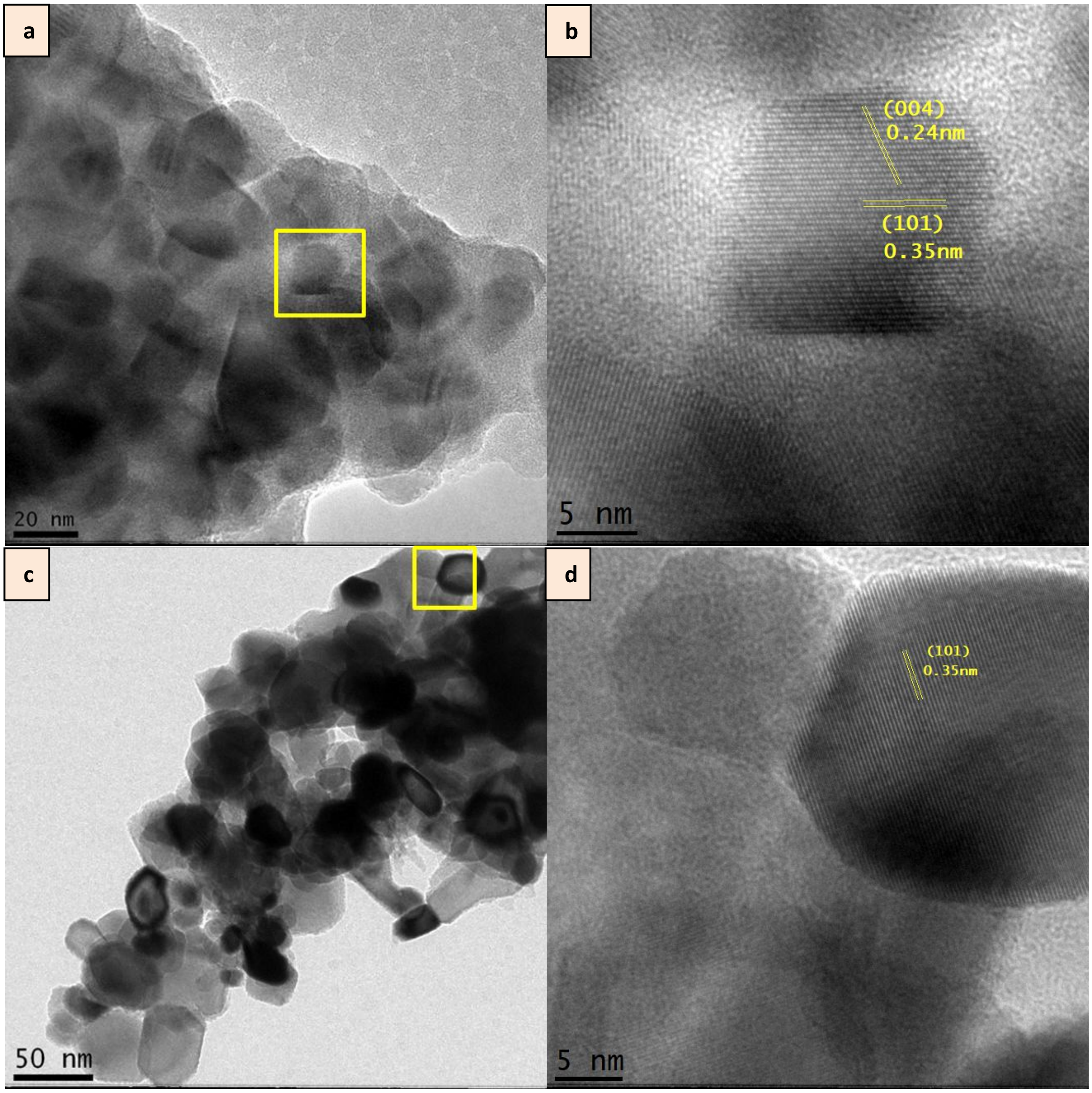

Figure S3: TEM micrographs of (a) SAP-TiO_2 at 50,000× magnification, (b) SAP-TiO_2 at 350,000× magnification, showing planes (004) and (101) of anatase phase, (c) SAP-AC/TiO_2 at 40,000× magnification, and (d) SAP-AC/TiO_2 at 400,000× magnification after MB adsorption, showing plane (101) of anatase phase.

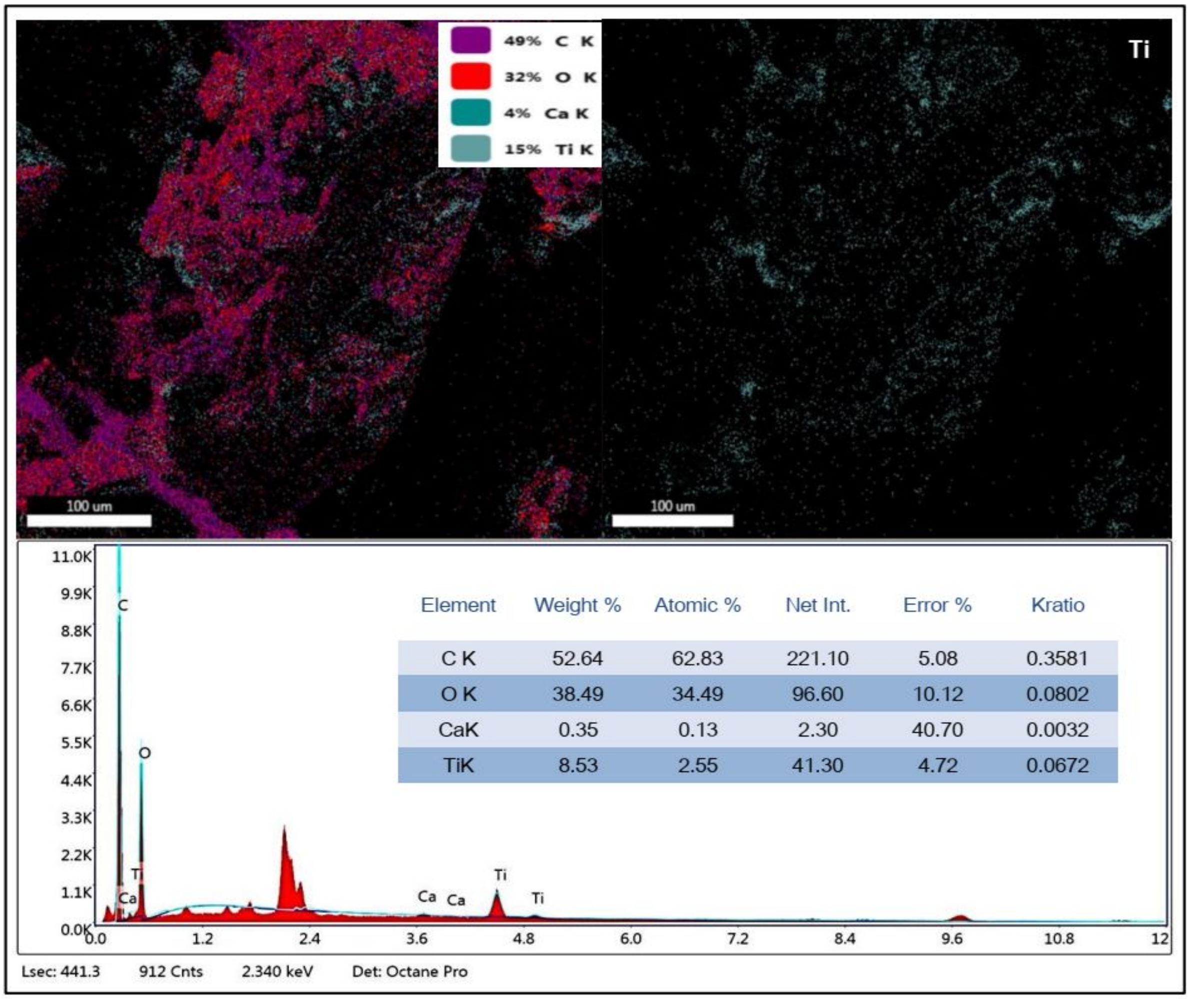

Element	Weight %	Atomic %	Net Int.	Error %	Kratio
C K	52.64	62.83	221.10	5.08	0.3581
O K	38.49	34.49	96.60	10.12	0.0802
CaK	0.35	0.13	2.30	40.70	0.0032
TiK	8.53	2.55	41.30	4.72	0.0672

Figure S4: EDS analysis of the spent SAP-TiO_2 composite after MB adsorption.

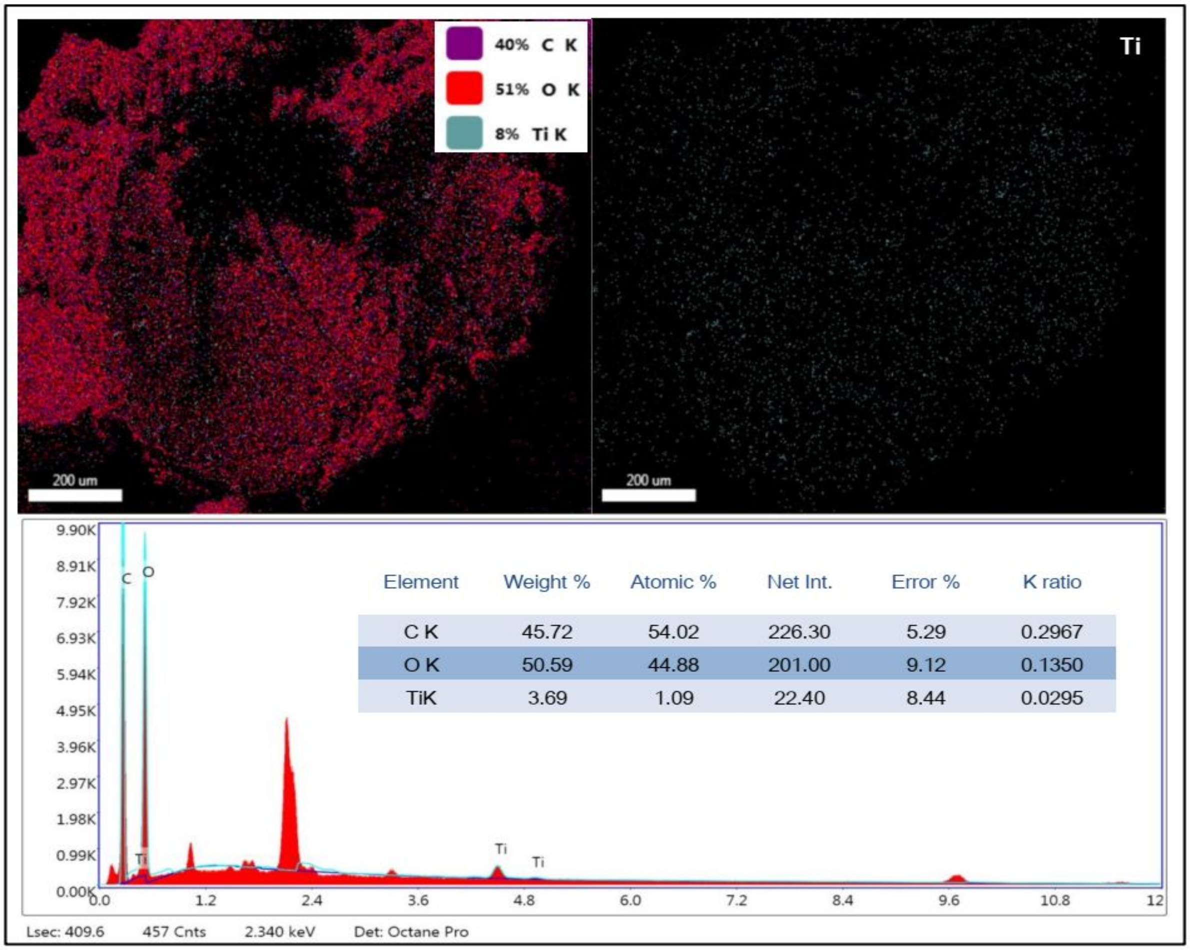

Figure S5: EDS analysis of the spent SAP-AC/TiO_2 composite after MB adsorption.

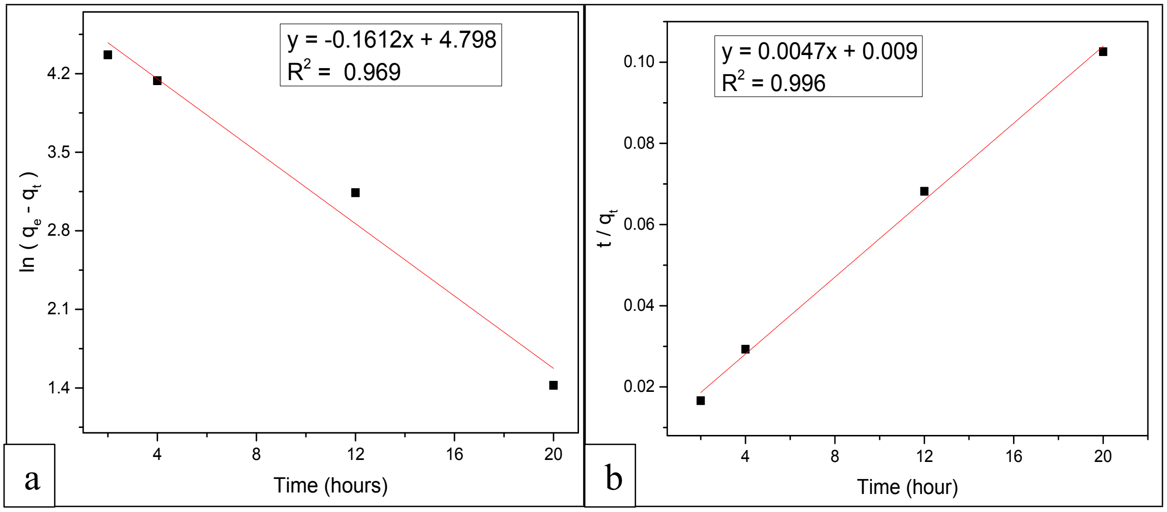

Figure S6: MB adsorption kinetics (a) pseudo first-order and (b) pseudo second-order using SAP-TiO_2 as adsorbent.

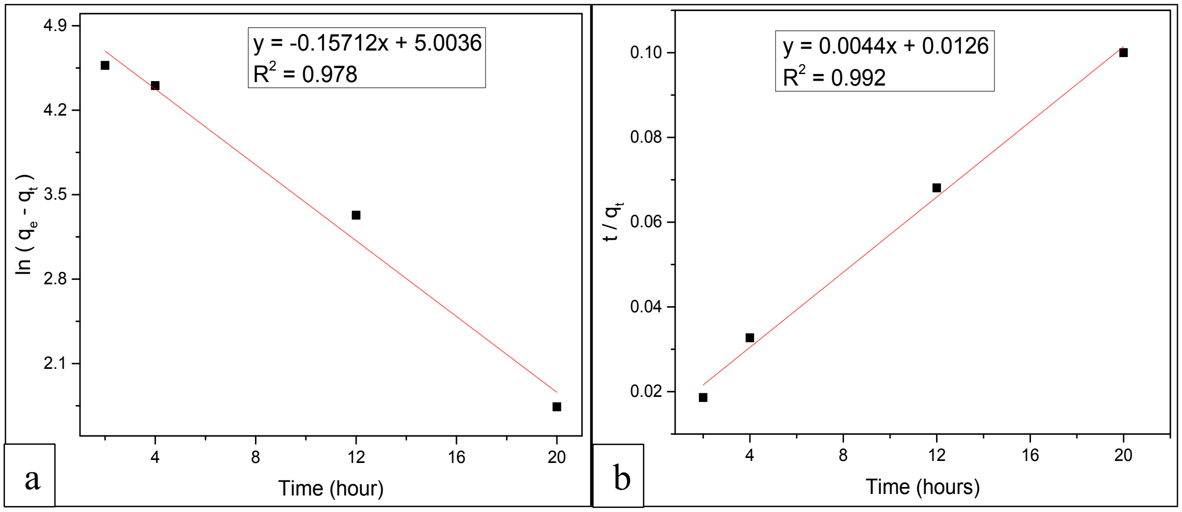

Figure S7: MB adsorption kinetics (a) pseudo first-order and (b) pseudo second-order using SAP-AC/TiO_2 as adsorbent.

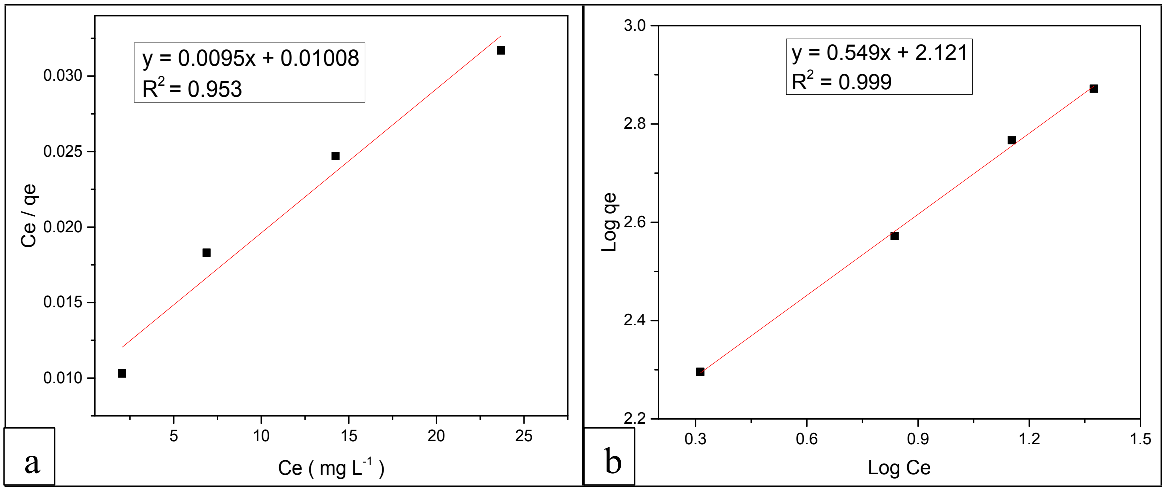

Figure S8: MB adsorption isotherms (a) Langmuir and (b) Freundlich model using SAP-TiO_2 as adsorbent.

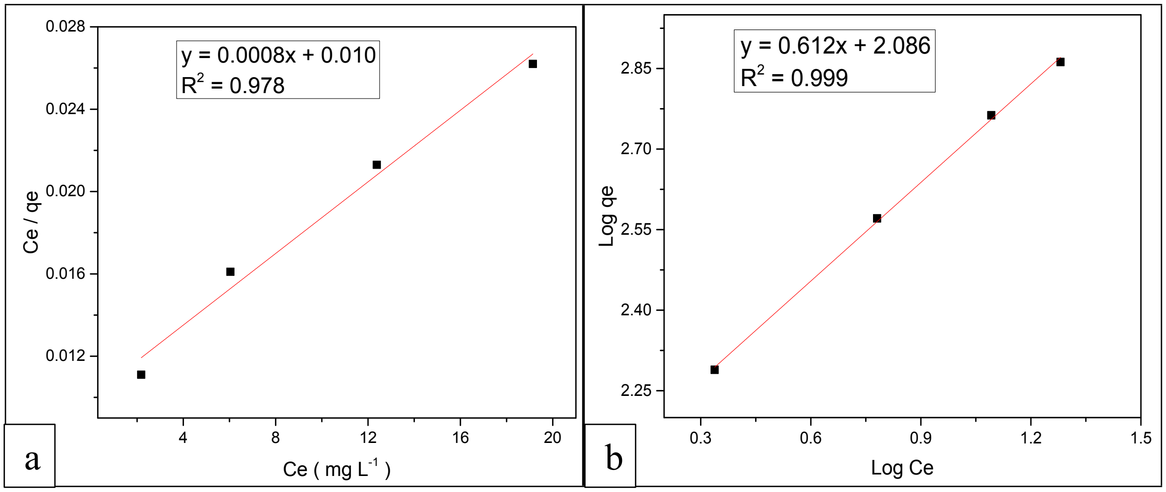

Figure S9: MB adsorption isotherms (a) Langmuir and (b) Freundlich model using SAP-AC/TiO_2 as adsorbent.

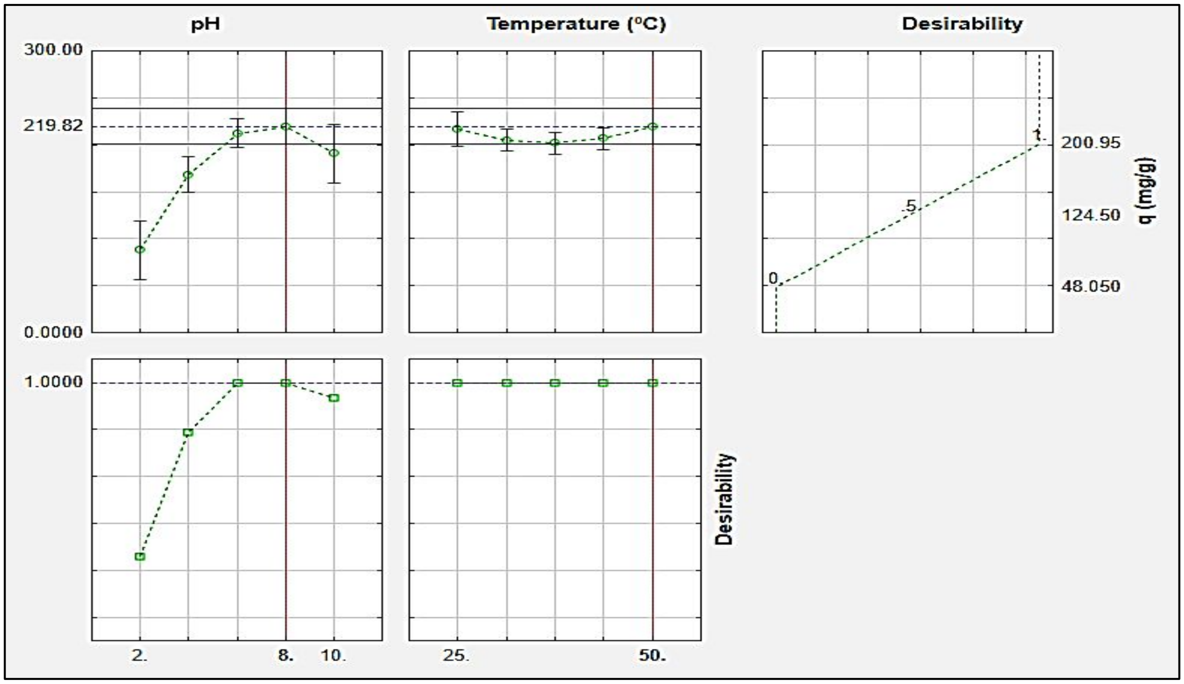

Figure S10: Desirability of MB adsorption at different pH and temperature conditions using SAP-TiO_2 as adsorbent.

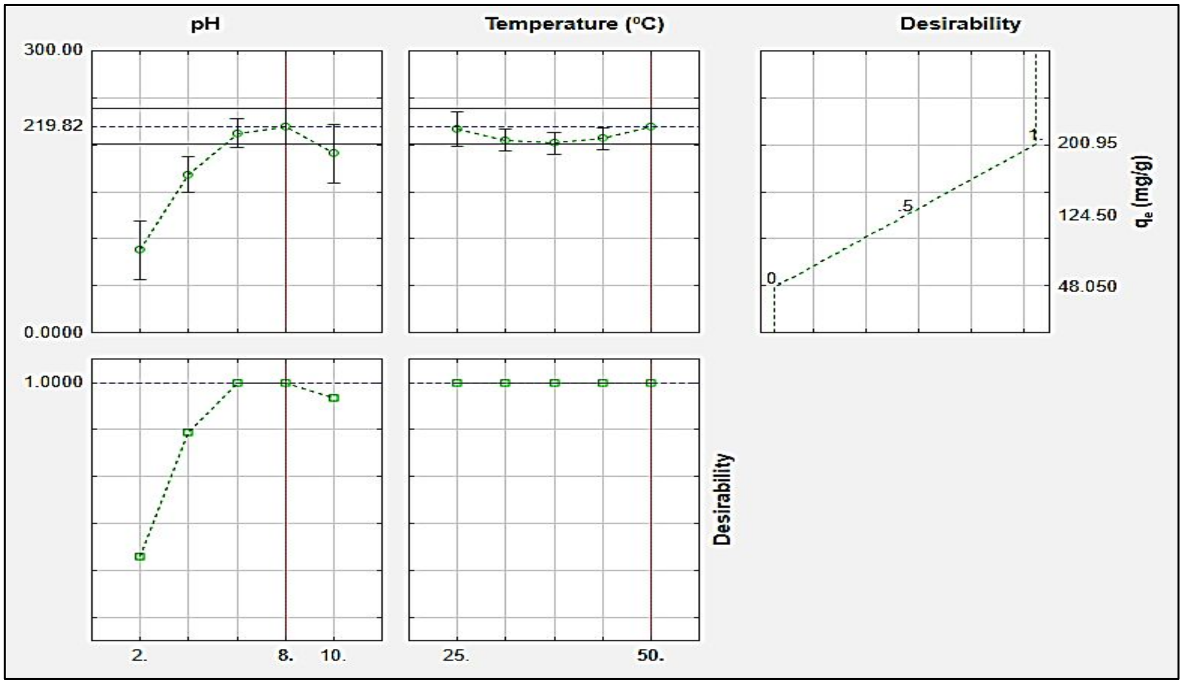

Figure S11: Desirability of MB adsorption at different pH and temperature conditions using SAP-AC/TiO_2 as adsorbent.

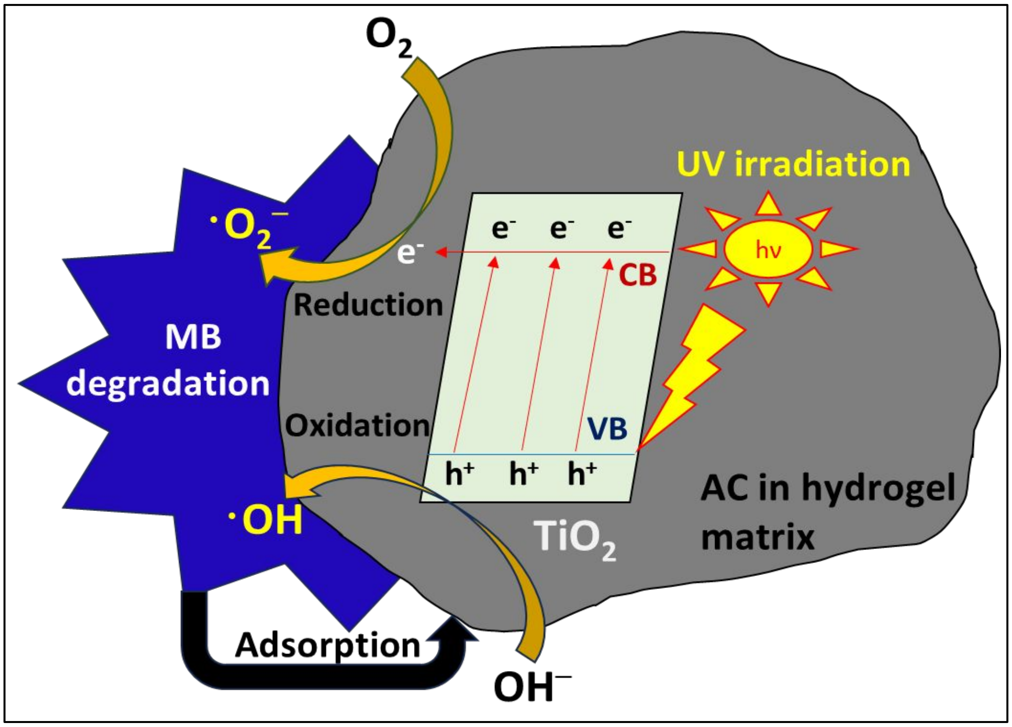

Figure S12: Schematic representation of the MB photocatalytic mechanism under UV irradiation.
